# On the nature of Co_*n*_^±/0^ clusters reacting with water and oxygen

**DOI:** 10.1038/s42004-024-01159-6

**Published:** 2024-03-30

**Authors:** Lijun Geng, Pengju Wang, Shiquan Lin, Ruili Shi, Jijun Zhao, Zhixun Luo

**Affiliations:** 1 Beijing National Laboratory for Molecular Sciences (BNLMS), State Key Laboratory for Structural Chemistry of Unstable and Stable Species, Institute of Chemistry, Chinese Academy of Sciences, Beijing, P. R. China; 2 Key Laboratory of Materials Modification by Laser, Ion and Electron Beams, Ministry of Education, Dalian University of Technology, Dalian, P. R. China; 3 University of Chinese Academy of Sciences, Beijing, P. R. China; 4 Guangdong Basic Research Centre of Excellence for Structure and Fundamental Interactions of Matter, Guangdong Provincial Key Laboratory of Quantum Engineering and Quantum Materials, School of Physics, South China Normal University, Guangzhou, P. R. China

**Keywords:** Chemistry, Nanoscience and technology, Reaction kinetics and dynamics, Electronic properties and materials, Information storage

## Abstract

Bulk cobalt does not react with water at room temperature, but cobalt nanometals could yield corrosion at ambient conditions. Insights into the cobalt cluster reactions with water and oxygen enable us to better understand the interface reactivity of such nanometals. Here we report a comprehensive study on the gas-phase reactions of Co_*n*_^±/0^ clusters with water and oxygen. All these Co_*n*_^±/0^ clusters were found to react with oxygen, but only anionic cobalt clusters give rise to water dissociation whereas the cationic and neutral ones are limited to water adsorption. We elucidate the influences of charge states, bonding modes and dehydrogenation mechanism of water on typical cobalt clusters. It is unveiled that the additional electron of anionic Co_*n*_^–^ clusters is not beneficial to H_2_O adsorption, but allows for thermodynamics- and kinetics-favourable H atom transfer and dehydrogenation reactions. Apart from the charge effect, size effect and spin effect play a subtle role in the reaction process. The synergy of multiple metal sites in Co_*n*_^–^ clusters reduces the energy barrier of the rate-limiting step enabling hydrogen release. This finding of water dissociation on cobalt clusters put forward new connotations on the activity series of metals, providing new insights into the corrosion mechanism of cobalt nanometals.

## Abstract (web summary)

Cobalt-based materials have a wide range of applications from information storage to electromagnetic absorbers in aerospace manufacturing, but the corrosion of cobalt nanosurfaces is poorly explored. Here, the authors combine mass spectrometry and DFT to study gas-phase reactions of Co_n_^±/0^ (n = 1–30) with water and oxygen and find that only anionic cobalt clusters give rise to water dissociation, whereas the cationic and neutral ones are limited to water adsorption.

## Introduction

As one of the three ferromagnetic metals in the periodic table of elements, cobalt is widely used in magnetic alloys with the advantage of heat resistance. Cobalt-based materials manifest a wide range of applications including permanent magnets^1^, information storage^2^ and aerospace manufacturing^3^. Since air and water affect the lifetime of these materials and the retention of their properties^4,5^, corrosion is an ever-present concern in the world of metals^6–8^. It is important to fully understand the interface interactions and reaction mechanism, which can guide the rational design of anticorrosion strategy for practical applications. The related metal–water interactions are also an important theme of research in chemistry and biology as well as energy source and environment^9,10^.

On the other hand, the low-cost and high-efficiency hydrogen production by water dissociation via electrolysis and photocatalysis is a long-term research topic^11–14^, for which cobalt nanocatalysts have attracted extensive interest, although cobalt usually does not react with water at ambient conditions^15–21^. Catalytic O–H dissociation and HAT is vital to water dehydrogenation and O-O bond formation^22–24^; however, catalytic oxidative dehydrogenation often exhibits limited activity and poor selectivity, despite decades of research efforts in this field. Small metal clusters possess distinct catalysis in contrast to their bulk analogues due to the quantum size effect and unique electronic structures^25^. For instance, dehydrogenation of water on some Al_*n*_^−^ clusters was observed at room temperature^26–28^, leading to the establishment of a complementary active site (CAS) mechanism^27^. Dehydrogenation of H_2_O molecules by reacting with gas-phase vanadium clusters was also noted^29^, showing diverse V_*n*_O^+^, V_*n*_O_2_^+^ and V_*n*_O_3_^+^ products by rapid reactions of V_*n≥3*_^+^ with water in a fishing mode^30^. In contrast to aluminium and vanadium, however, cobalt does not support water dehydrogenation according to the activity series of metals^31^. There comes a pending question if subnanometer cobalt clusters can support spontaneous water dehydrogenation and oxidation, which leads to a better understanding of the corrosion of cobalt nanosurfaces.

Based on this motivation, herein we report a comprehensive study of the gas-phase reactions of Co_*n*_^±/0^ clusters with water and oxygen. Well-resolved Co_*n*_^±/0^ (c.a., *n* = 1–30) clusters are prepared, and their reactions with water are studied by using our self-developed ultrafast deep ultraviolet laser ionisation mass spectrometer (DUV-LIMS, Supplementary Fig. S1)^32,33^. As a result, we found all these Co_*n*_^±/0^ clusters react with oxygen to form diverse oxides. However, the Co_*n*_^+^ clusters readily react with water giving rise to diverse adsorption products, which contrasts with the anionic Co_*n*_^–^ clusters which allow for dehydrogenation in reacting with water. Combined with density functional theory (DFT) calculations, we illustrated the reaction dynamics and unveiled the altered binding mode of water on the small Co_*n*_^–^ cluster anions (Co_*n*_^–^···H–OH) compared with their cationic and neutral analogues (Co_*n*_^+/0^·OH_2_). Apart from the charge effect, we also elucidated the spin effect and cooperative multi-site effect that promote water dissociation and dehydrogenation on the Co_*n*_^–^ clusters, showing enhanced activity of such cobalt clusters without being restricted by the principles of activity series of metals.

## Results and discussion

### Anionic Co_*n*_^–^ clusters reacting with water

**Fig. 1:**
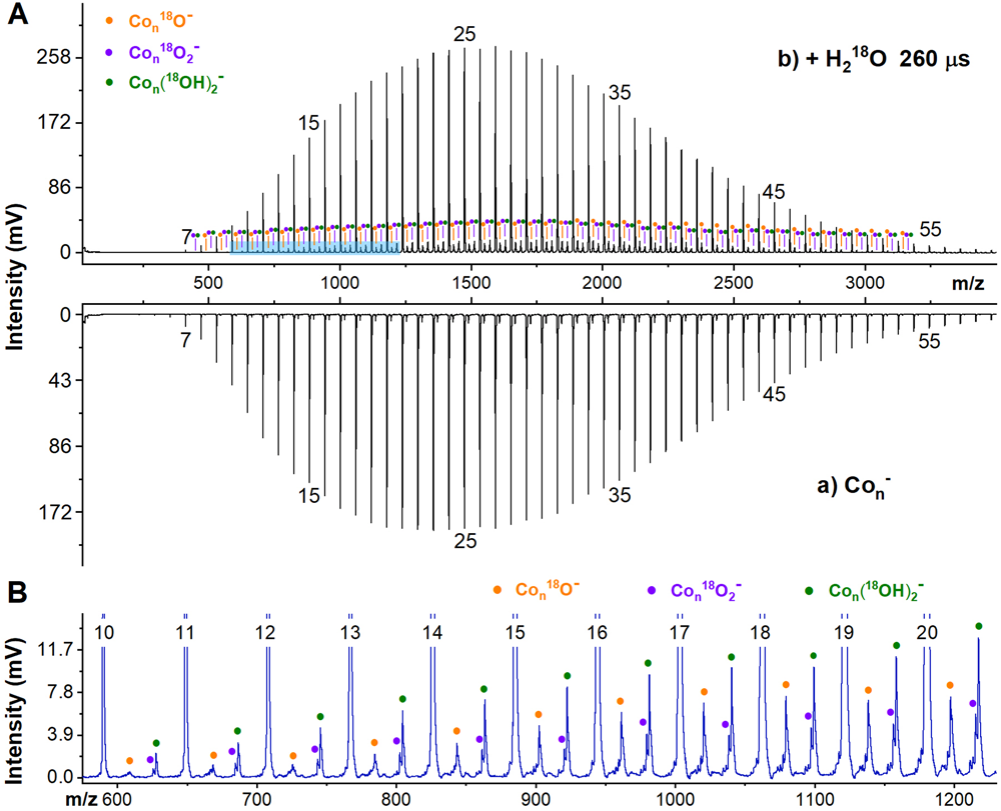
Typical mass spectrum of Co_*n*_^–^ reacting with H_2_^18^O. **A** Typical mass spectra of the Co_*n*_^–^ clusters produced by the homemade LaVa source, within a 35 mm nozzle and 10.0 atm He buffer gas, and the reaction products with H_2_^18^O being introduced into the flow tube, controlled by a pulsed valve with a pulse width at 260 µs. **B** Enlarged area for the Co_*n*_^–^ (*n* = 10–20) clusters after the reaction with H_2_^18^O.

The reactions of Co_*n*_^±/0^ clusters with oxygen have been addressed in our previous study^34^, showing a tendency to form diverse oxides (Supplementary Fig. S2) but with a stable cluster Co_13_O_8_ showing up in the presence of sufficient oxygen reactant. Here we emphasize on the reactions with water. Figure 1A presents a typical mass spectrum of anionic Co_*n*_^–^ (*n* = 5–59) clusters in the absence and presence of water carried by bubbling of He buffer gas. The prepared Co_*n*_^–^ clusters display a regular Gaussian/Rayleigh distribution centred at Co_25_^–^. Isotope-labelled water, H_2_^18^O, was used to exclude the interference of trace amount of oxygen contamination (also, deuterium water D_2_O was also used to unambiguously identify the dehydrogenation products, Supplementary Fig. S3). Figure 1B displays an enlarged area to visualise the products of Co_*n*_^–^ (*n* = 10–20) clusters reaction with H_2_^18^O. The observation of a series of products [Co_*n*_^18^O]^–^, [Co_*n*_^18^O_2_]^–^ and [Co_*n*_ (^18^OH)_2_]^–^ suggests that the Co_*n*_^–^ clusters undergo dehydrogenation with one and two water molecules.

**Fig. 2:**
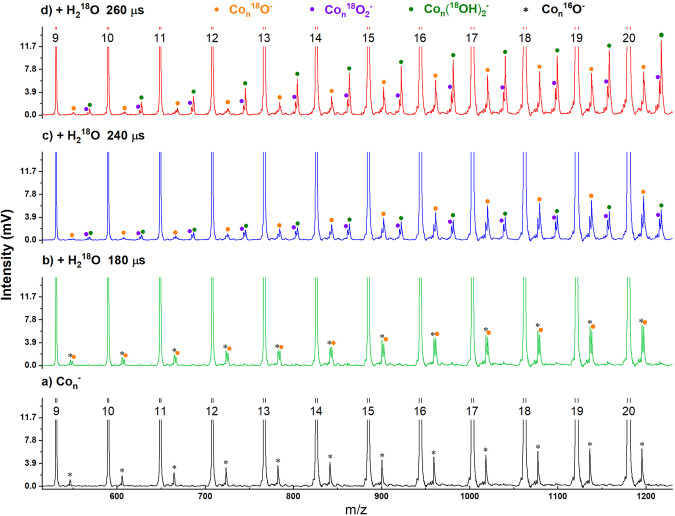
Mass spectrum of Co_*n*_^–^ reacting with H_2_^18^O of different doses. **a** Mass spectra of the Co_*n*_^–^ clusters. **b**–**d** Mass spectra of Co_*n*_^–^ clusters after reactions with different amounts of H_2_^18^O, controlled by a pulsed valve with varying pulse widths at 180 µs, 240 µs, and 260 µs, respectively, corresponding to the original Supplementary Fig. S4. The peaks marked with stars (*) correspond to oxygen attachment due to the trace amount of contamination.

We monitored the reaction of Co_*n*_^–^ clusters with water at varying doses of the water controlled by the pulsed valve (Fig. 2 and Supplementary Fig. S4). An estimation of reaction rates is given in Supplementary Fig. S5. When the cobalt clusters reacted with small amounts of water, a series of [Co_*n*_^18^O]^–^ products (accompanied by minor Co_*n*_O^–^ contamination) were observed in the mass spectra. As the dose of water was gradually increased, the [Co_*n*_ (^18^OH)_2_]^–^ and [Co_*n*_^18^O_2_]^–^ products appeared in the mass spectra (and the nascent Co_*n*_O^–^ contamination peaks disappeared). When a large amount of water was involved in the reaction, a series of [Co_*n*_(^18^OH)_2_]^–^ products dominated the mass spectra. Interestingly, the adsorption products [Co_*n*_H_2_^18^O]^–^ and [Co_*n*_(H_2_^18^O)_2_]^–^ were absent in the mass spectrometry observation, indicating that the HAT and dehydrogenation proceeded rapidly. This was also verified by the experiments based on deuterium water (D_2_O, Supplementary Fig. S3). This experimental observation challenges the previously established principles that cobalt reacts with protonic acid (but not H_2_O)^31^ to form H_2_. The dehydrogenation reactions of anionic cobalt clusters with water can be written as,

1 $${{{{{{\rm{Co}}}}}}}_{n}^{{{-}}}+{{{{{{\rm{H}}}}}}}_{2}{{{{{\rm{O}}}}}}\to {{{{{{\rm{Co}}}}}}}_{n}{{{{{{\rm{O}}}}}}}^{{{-}}}+{{{{{{\rm{H}}}}}}}_{2}$$

2 $${{{{{{\rm{Co}}}}}}}_{n}^{{{-}}}+{2{{{{{\rm{H}}}}}}}_{2}{{{{{\rm{O}}}}}}\to {{{{{{\rm{Co}}}}}}}_{n}{({{{{{\rm{OH}}}}}})}_{2}^{{{-}}}+{{{{{{\rm{H}}}}}}}_{2}$$

3 $${{{{{{\rm{Co}}}}}}}_{n}^{{{-}}}+2{{{{{{\rm{H}}}}}}}_{2}{{{{{\rm{O}}}}}}\to {{{{{{\rm{Co}}}}}}}_{n}{{{{{{\rm{O}}}}}}}_{2}^{{{-}}}+2{{{{{{\rm{H}}}}}}}_{2}$$

### The reactions of neutral and cationic Co clusters

**Fig. 3:**
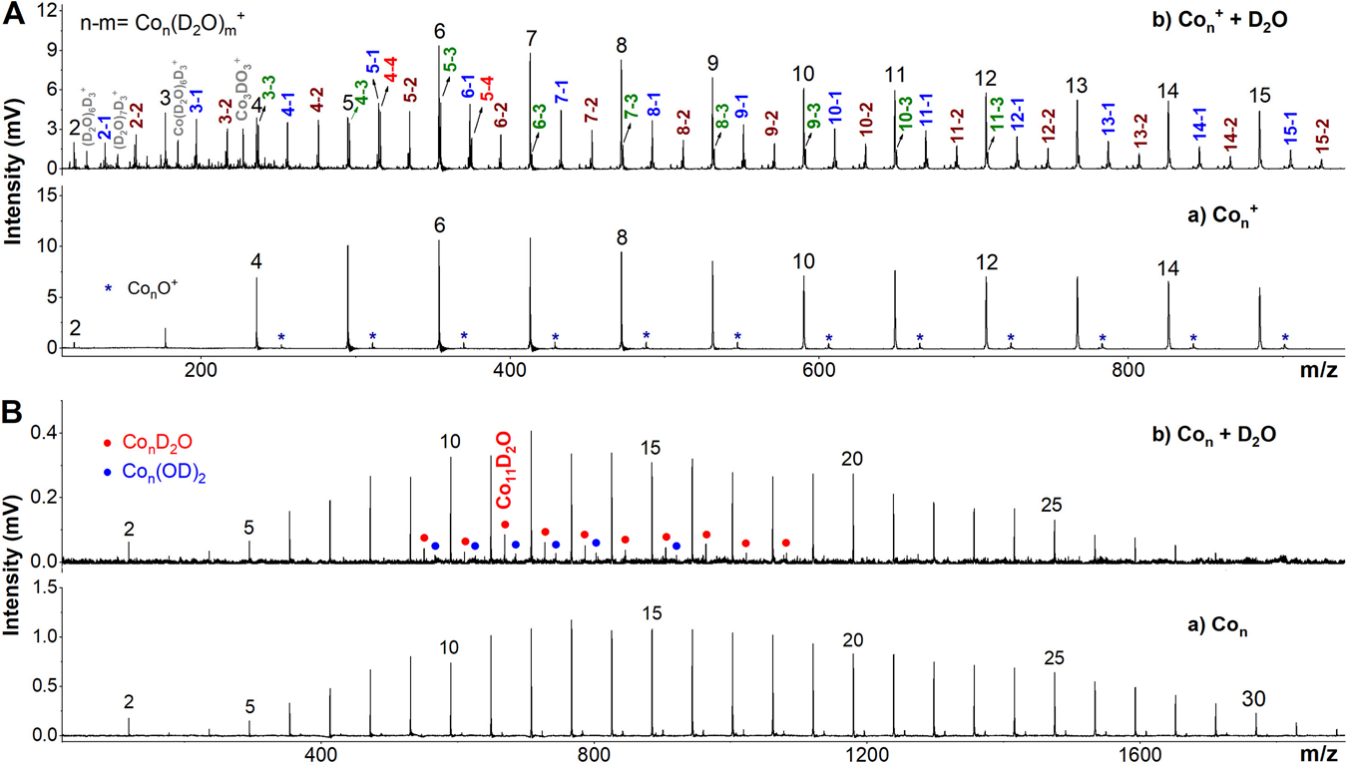
Co_*n*_^+,0^ reacting with D_2_O. **A** Typical mass spectra of the cationic Co_*n*_^+^ (*n* = 2–15) clusters before and after reacting with D_2_O in the flow tube. **B** Typical mass spectra of the neutral Co_*n*_ (*n* = 2–30) clusters before and after reacting with D_2_O.

To compare the reaction behaviour of cobalt cluster anions, neutrals and cations, Fig. 3 displays the mass spectra of the cobalt cluster cations and neutrals before and after reacting with water, respectively. To distinguish the likely hydrogenation products, the isotope chemical D_2_O was used. As a result, the cationic Co_*n*_^+^ clusters were found to adsorb multiple D_2_O molecules showing diverse Co_*n*_^+^(D_2_O)_*m*_ complexes; however, almost no dehydrogenation products were observed except for Co_3_^+^. The observation of strong water adsorption on Co_*n*_^+^ clusters is consistent with the previous studies of Rh_*n*_^+^ clusters reacting with water, as well as the Co_*n*_^+^ clusters reacting with NH_3_ (ref. ^35^). Natural bond orbital (NBO) analysis shows maximal donor–acceptor orbital overlap interaction energy between the cationic clusters and H_2_O molecule (Supplementary Fig. S12), which is also in agreement with the results of charge decomposition analysis and potential scan for a H_2_O molecule in approaching a cobalt cluster (Supplementary Figs. S16 and S17). There is a similar case for the neutral cobalt clusters which also exhibit weak reactivity with water, with a few water-adsorption products being observed, such as Co_9-18_D_2_O (Fig. 3B). The different reactions of Co_*n*_^±/0^ clusters with water embody the charge dependence of metal cluster reactivity, as revealed in the previous studies on the reactivities of Al, Nb and Rh clusters^36–39^.

### Reaction dynamics and charge effect

We have conducted DFT calculations to elucidate the charge effect and H_2_ release mechanism of cobalt clusters in reacting with water molecules. The structures of Co_*n*_^±/0^ and [Co_*n*_H_2_O]^±/0^ (*n* = 2–13) clusters with different spin multiplicities are optimised at the PBE-D3/def2-TZVP level of theory (Supplementary Figs. S7–11 and Table S1). Interestingly, the lowest-energy structures of water adsorption on the cationic and neutral clusters prefer Co–O coordination (i.e., forming [Co_*n*_OH_2_]^+/0^), with slight fluctuation of the bond lengths (Supplementary Fig. S14); however, water adsorption on the Co_*n*_^−^ (*n* = 1, 2, 3, 5, 6) clusters results in Co–H bonding (Co_*n*_^–^···H–OH). This is consistent with the previous study of M(H_2_O)^–^ (M = Cu, Ag, Au)^40^. In addition, the binding energies (*E*_ad_) of H_2_O onto the Co_*n*_^±/0^ clusters show significant charge dependence. The *E*_ad_ values of the cations are larger than those of the neutral and anionic clusters (Supplementary Fig. S13), in line with the experimental observation that the cations can adsorb multiple water molecules while the reaction products of the anions are relatively small, although they support H_2_ release.

**Fig. 4:**
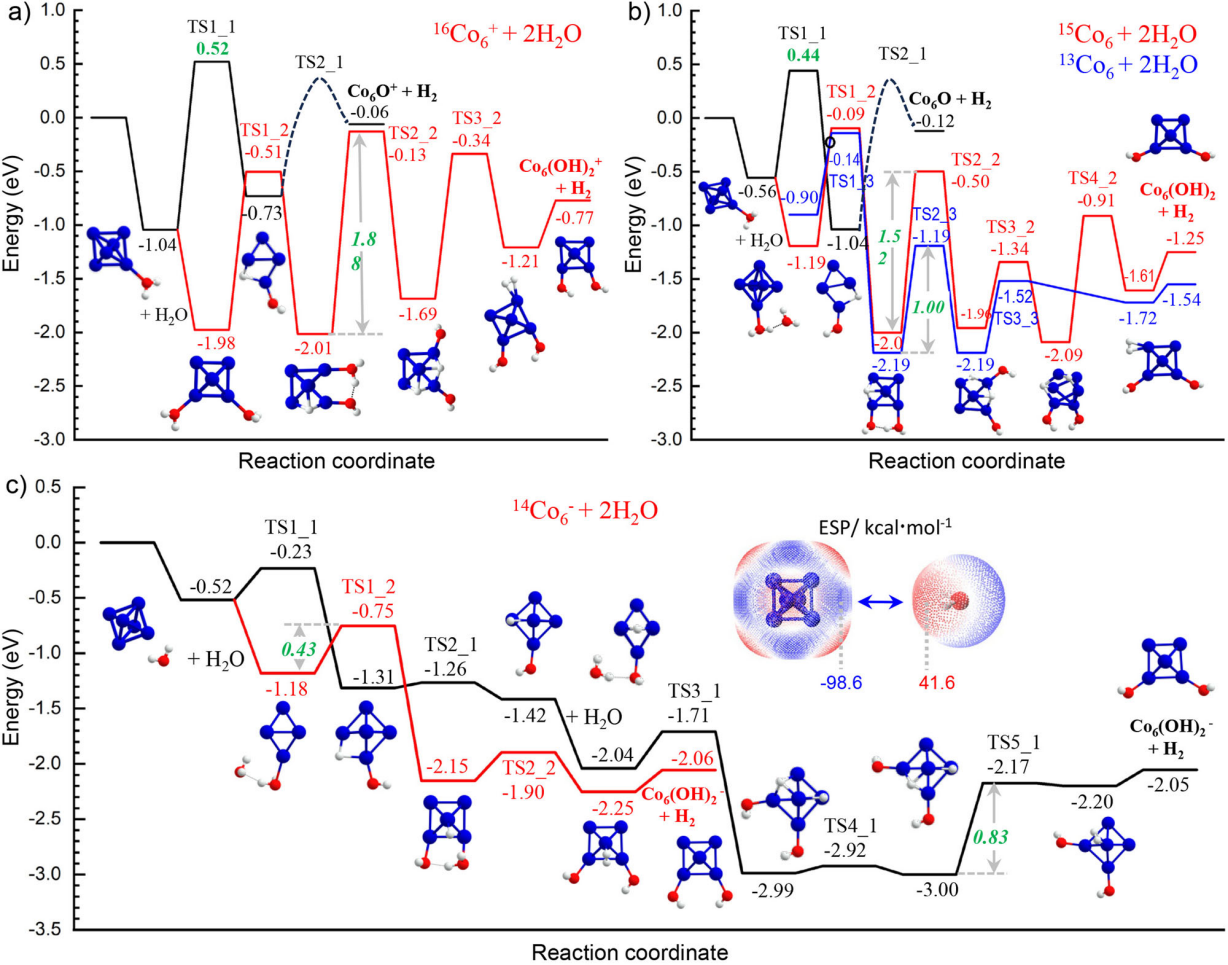
Reaction coordinates of Co_6_^±/0^. **a**–**c** The energy diagram for cationic ^16^Co_6_^+^, anionic ^14^Co_6_^−^, and neutral ^15^Co_6_ clusters in reacting with one and two water molecules. Energies are given in eV. The inset shows electrostatic potentials (EPS, kcal mol^−1^) for a H_2_O molecule in approaching the Co_6_^−^ cluster with spontaneously regulated orientation.

We carried out DFT calculations on the thermodynamic energies and reaction kinetics typically for one and two H_2_O molecules to react with Co_6_^±/0^ which has an octahedral structure. As shown in Fig. 4, both Co_6_ and Co_6_^+^ clusters suffer from unsurmountable energy barriers of the H-atom transfer (TS1_1 at 0.52 eV and 0.44 eV higher than the reactants); in contrast, HAT on the Co_6_^−^ cluster is thermodynamically favourable and kinetically favourable with a small energy barrier. While for the reaction of “Co_6_^±/0^ + 2 H_2_O”, the reaction coordinates indicate that three reactions are exothermic, but the energy for the H-atom transfer step (TS1_2) still differs from each other, and the neutral and cationic clusters take on larger single-step energy barriers. In addition, the reaction pathways for the cationic and anionic Co_6_^±^ clusters obey spin conservation, but the energy barrier of the rate-determining step for the Co_6_^−^ cluster (0.43 eV) is much lower than that of the Co_6_^+^ (1.88 eV). Also, for the reaction of neutral Co_6_ with two H_2_O molecules, the pathway of spin conservation (red-curve) suffers from a higher energy barrier of TS2_2 (1.52 eV); in comparison, the blue-curve pathway begins with a lower spin adsorption state (^13^Co_6_) and undergoes a relatively lower energy barrier (1.00 eV) of the rate-determining step. Although the spin crossing causes a reduced energy barrier, the dehydrogenation on neutral Co_6_ is still not favourable compared with the anionic Co_6_^−^. It can be concluded that both spin states and charge effect play a dramatic role in the catalytic dehydrogenation on such nanometals^41^. Notably, the cool He buffer gas could take away part of the energy during the reaction, rendering the cationic and neutral clusters not having enough energy for dehydrogenation (Supplementary Tables S2 and S3), especially for those having large energy barriers of the transition states.

### Size effect and multi-site cooperation

**Fig. 5:**
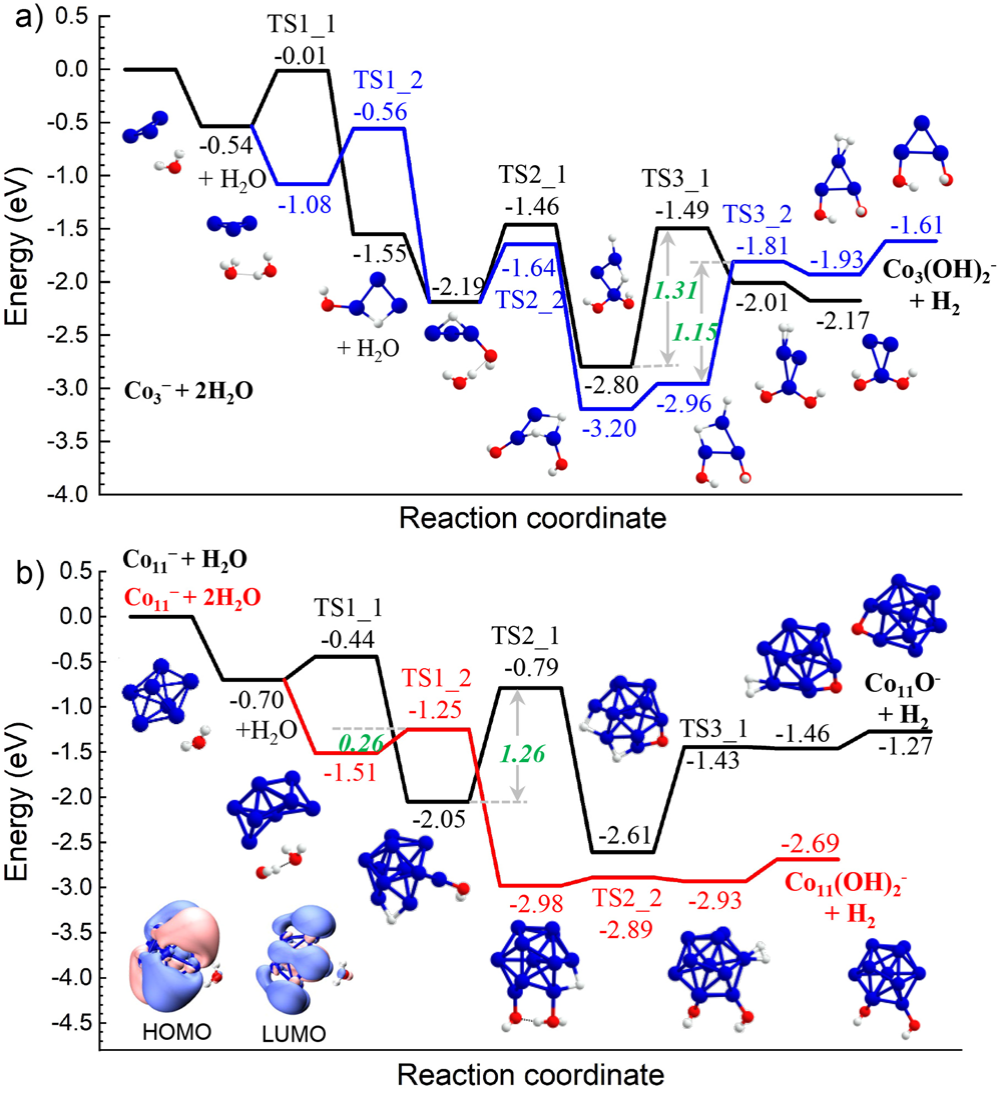
A comparison of Co_3_^−^ with Co_11_^−^. **a** Reaction energy diagram of “Co_3_^− ^+ 2 H_2_O → Co_3_(OH)_2_^− ^+ H_2_”. **b** Reaction energy diagram of “Co_11_^− ^+ H_2_O → Co_11_O^− ^+ H_2_” and “Co_11_^− ^+ 2 H_2_O → Co_11_(OH)_2_^− ^+ H_2_”. Energies are given in eV. The insets show the corresponding structures. The inset on the left bottom shows the HOMO and LUMO patterns of [Co_11_H_2_O]^−^.

According to our DFT calculations, the reactions of Co_3_^−^ and Co_11_^−^ are also initiated by hydrogen-metal bonding adsorption (Fig. 5), similar to the aforementioned Co_6_^−^ in reacting with two H_2_O molecules. Notably, the first H-atom transfer of a single H_2_O on the Co_3_^−^ cluster is thermodynamically unfavourable. This is different from the previous finding of V_*n*≥3_^−^ clusters in reacting with a single water molecule to release H_2_, which is associated with the nature of the metal activity sequence. Nevertheless, Co_3_^−^ reacts with two H_2_O molecules to release H_2_, shedding light on the importance of synergetic active sites and multiple molecule cooperation.

In comparison, the reaction of Co_11_^−^ with a single H_2_O finds a relatively larger energy gain of the adsorption and smaller energy barrier for the first H-atom transfer; nevertheless, the final transition state of H-H recombination for H_2_ evolution displays comparable single-step energy barrier as the Co_3_^−^. Notably, the reaction of “Co_11_^− ^+ 2 H_2_O → Co_11_(OH)_2_^− ^+ H_2_” shows a much smaller energy barrier (0.26 eV) for the H-atom transfer (TS1_2) compared with the rate-determining step for a complete dehydrogenation (1.34 eV for TS3_2, Supplementary Fig. S18). This coincides with the experimental observation of a larger mass abundance of Co_*n*_(OH)_2_^−^ than Co_*n*_O_2_^−^.

### The reactions of both water and oxygen

**Fig. 6:**
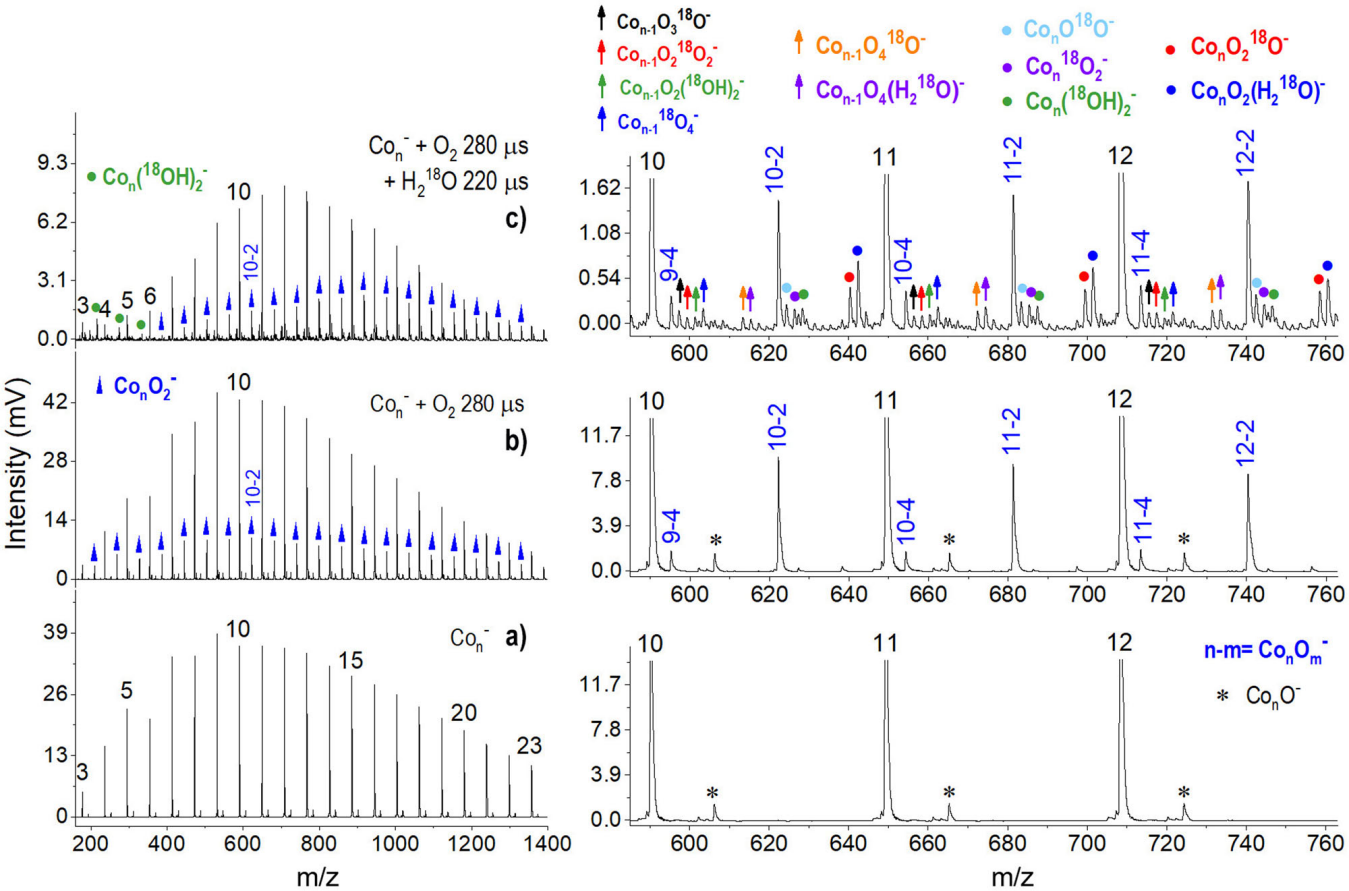
Co_*n*_^−^ reacting with O_2_ and D_2_O. **a** Typical mass spectrum of the anionic Co_*n*_^−^ (*n* = 3–23) clusters. **b** The mass spectrum after their reactions with oxygen (1% in He). **c** The mass spectrum after reactions with both O_2_ and H_2_^18^O in the flow tube, controlled by two pulse valves. The enlarged mass ranges for Co_10-12_^−^ are given on the right side, respectively.

Considering that the corrosion of metals is essentially related to their chemical reaction with oxygen and water to form oxidation and dehydrogenation products, we further studied the reactions of the anionic Co_*n*_^−^ clusters by introducing both water and oxygen as reactants into the flow tube. The results are given in Fig. 6. It is seen that oxygen reacts with the Co_*n*_^−^ clusters to form Co_*n*_O_2*x*_^−^ clusters without exception but with slightly lower reaction rates at Co_5_^−^ and Co_6_^−^, likely due to their structural stability^5^. Meanwhile, partial dehydrogenation products, including a series of [Co_*x*_O_*y*_·^18^O]^−^, [Co_*x*_O_*y*_·^18^O_2_]^−^ and [Co_*x*_O_*y*_(^18^OH)_2_]^−^ were observed, indicating that oxygen undergoes competitive adsorption but does not hinder dehydrogenation. Nevertheless, from the diverse products of oxidation and dehydrogenation, it can be inferred that nanoscale cobalt suffers from inevitable corrosion, although this reactivity could not be so fast as iron. By referring to the corrosion equation of iron^42^, the reactivity of cobalt clusters with water and oxygen could be summarised by an integrated reaction channel,

4 $${{{{{\rm{C}}}}}}{{{{{{\rm{o}}}}}}}_{n}^{{{-}}}	 +\, x{{{{{{\rm{H}}}}}}}_{2}{{{{{\rm{O}}}}}}+y{{{{{{\rm{O}}}}}}}_{2}\to {{{{{{\rm{Co}}}}}}}_{n}{{{{{{\rm{O}}}}}}}_{m}{({{{{{\rm{OH}}}}}})}_{u}{({{{{{\rm{H}}}}}}_{2}{{{{{\rm{O}}}}}})}_{v}^{{{-}}}\\ 	 + \, (x-v-u/2){{{{{{\rm{H}}}}}}}_{2},x+2y=m+u+v$$

We would like to put forward more discussion on the corrosion mechanism of nano-cobalt. Under ambient moist and oxygen-rich atmosphere, most metals suffer from spontaneous oxidation and likely thermodynamical dehydrogenation with few exceptions (gold and platinum)^43^. Some metals such as aluminium, chromium, magnesium and nickel can be well protected by a layer of impenetrable oxide coatings that prevents further destruction of the surface. So does the stainless steel which usually involves chromium and nickel to attain dense protection, thus avoiding unwanted corrosion. Whether or not a tight protective film, it is vital to avoid the formation of hydrated metal oxide and prevent the metal rusts from continually flaking off, without an exposure of fresh metal surfaces to oxygen and water. According to our results, it could be helpful to avoid negative charge accumulation; in other words, it would be important to check surface static charge regularly and keep neutral surfaces. In addition, a previous study found that Co_13_O_8_ is a highly stable cluster oxide^34^, which could also breed a strategy of tight protective film like Al_2_O_3_.

### Conclusions

In summary, we report a joint experimental and theoretical study of cobalt clusters Co_*n*_^±/0^ in reacting with water and oxygen. All the Co_*n*_^±/0^ clusters were found to react with oxygen regardless of the presence of water or not. However, water dissociation is observed only for the anionic Co_*n*_^–^ (*n* = 5–59) clusters, but the cationic Co_*n*_^+^ and neutral Co_*n*_ clusters do not support the observation of dehydrogenation except for Co_3_^+^. Combined with the DFT results, we unveil the bonding mechanisms of the Co_*n*_^±/0^ clusters and illustrate the reaction kinetics of typical Co_*n*_^–^ clusters toward water in forming Co_*n*_O^–^, Co_*n*_(OH)_2_^–^, and Co_*n*_O_2_^–^ products. Notably, the cobalt catalysis for dehydrogenation processes is not inhibited in the presence of oxygen; instead, a series of products of oxidation and partial dehydrogenation embody the corrosion of nano-cobalt surfaces.

## Methods

### Experimental methods

The instrumentation used in this study is based on a customised reflection time-of-flight mass spectrometer (Re-TOFMS). Detailed descriptions can be found in our previous publications^33,35,44^. In brief, the Re-TOFMS is equipped with a flow tube reactor which is connected with dual pulse valves enabling reactions with two reactant gases (e.g., O_2_ and water). Isotopic chemicals of both D_2_O and H_2_^18^O were used to help identify the reaction products. The Co_*n*_^±^ clusters were prepared by ablating a clean cobalt disk ($\varPhi$ = 16 mm, 99.95%) with a pulsed laser (10 Hz 532 nm Nd: YAG) in the presence of helium buffer gas (99.999%, 10.0 atm). The Co_*n*_^±^ clusters were prepared and ejected out of a nozzle ($\varPhi$ = 2 mm, *L* = 35 mm) during a supersonic expansion process controlled by the pulsed valve (Series 9, General Valve). For reactions between the cobalt clusters and water (D_2_O and H_2_^18^O), water vapour was injected into the flow tube reactor ($\varPhi$ = 6 mm, *L* = 60 mm) by the He (99.999%, 1 atm) bubbling method. Oxygen reactant was diluted (1% in helium) and introduced from the other pulse valve connected to the same flow tube reactor. The reactants were controlled by varying the on-time pulse width. All metal clusters and their reaction products were detected and analysed by the Re-TOFMS. For the neutral Co_*n*_ clusters reacting with D_2_O, we used an all-solid-state deep ultraviolet (DUV) laser (177.3 nm wavelength, 15.5 ps pulse width, 10 Hz repletion rate, and ∼15 μJ energy per pulse) with a head-to-head mode in the ionisation zone.

### DFT calculation methods

The DFT calculations were performed with the PBE-D3 corrected functional^45^ using the Gaussian 16 programme^46^. The geometric optimisation and reaction coordinate research were carried out using the balanced triple-zeta def2-TZVP basis set^47^ for Co, O and H atoms. Vibrational frequency calculations were carried out to ensure that the lowest-energy structures of reaction products have no imaginary frequencies and the transition states (TSs) have only one imaginary frequency. All energies were corrected with zero-point vibrations and the intrinsic reaction coordinate (IRC) scan was employed to ensure a connection with both intermediates in the reaction pathway. The natural bond orbital (NBO), electrostatic potential (ESP), and charge decomposition analysis were analysed by Multiwfn software^48^. Orbitals and ESP patterns were drawn by the visual molecular dynamics (VMD) software^49^.

### Supplementary information

Peer Review File

Supplementary Information

## Data Availability

The data that support the findings of this study are available within the article and its Supplementary Information or from the corresponding author upon reasonable request.

## References

[1] Balasubramanian B (2011). Cluster synthesis and direct ordering of rare-earth transition-metal nanomagnets. Nano Lett..

[2] Thurn-Albrecht T (2000). Ultrahigh-density nanowire arrays grown in self-assembled diblock copolymer templates. Science.

[3] Zhou J (2023). Interfacial electronic heterostructure engineering of cobalt boride nanosheets toward broadband efficient electromagnetic absorption. Chem. Eng. J..

[4] Lentijo-Mozo S (2015). Air- and water-resistant noble metal coated ferromagnetic cobalt nanorods. ACS Nano.

[5] Geng L (2021). Reactivity of cobalt clusters Co_n_^±/0^ with dinitrogen: superatom Co_6_^+^ and superatomic complex Co_5_N_6_^+^. J. Phys. Chem. A.

[6] Carrasco J, Hodgson A, Michaelides A (2012). A molecular perspective of water at metal interfaces. Nat. Mater..

[7] Jia Y (2021). Interactions between water and rhodium clusters: molecular adsorption versus cluster adsorption. Nanoscale.

[8] Taylor, C. D., Gale, J. D., Strehblow, H. H. & Marcus, P. In *Molecular Modeling of Corrosion Processes* (eds Taylor, C. D. et al.) 1−34 (John Wiley & Sons, 2015).

[9] Kiawi DM (2015). Water adsorption on free cobalt cluster cations. J. Phys. Chem. A.

[10] Castro M (2012). Theoretical study of negatively charged Fe^–^(H_2_O)_N ≤ 6_ clusters. J. Phys. Chem. A.

[11] Turner JA (2004). Sustainable hydrogen production. Science.

[12] Wang B (2023). Homogeneous pseudoamorphous metal phosphide clusters for ultra stable hydrogen generation by water electrolysis at industrial current density. Chem. Eng. J..

[13] Wang J (2016). Recent progress in cobalt-based heterogeneous catalysts for electrochemical water splitting. Adv. Mater..

[14] Chen R (2021). Integration of bio-inspired lanthanide-transition metal cluster and p-doped carbon nitride for efficient photocatalytic overall water splitting. Natl Sci. Rev..

[15] Kanan MW, Nocera DG (2008). In situ formation of an oxygen-evolving catalyst in neutral water containing phosphate and Co^2+^. Science.

[16] Fei H (2015). Atomic cobalt on nitrogen-doped graphene for hydrogen generation. Nat. Commun..

[17] Mattioli G, Giannozzi P, Amore Bonapasta A, Guidoni L (2013). Reaction pathways for oxygen evolution promoted by cobalt catalyst. J. Am. Chem. Soc..

[18] Gopi S, Giribabu K, Kathiresan M, Yun K (2020). Cobalt(Ii) ions and cobalt nanoparticle embedded porous organic polymers: an efficient electrocatalyst for water-splitting reactions. Sust. Energ. Fuels.

[19] Mao J (2018). Accelerating water dissociation kinetics by isolating cobalt atoms into ruthenium lattice. Nat. Commun..

[20] Du P, Eisenberg R (2012). Catalysts made of earth-abundant elements (Co, Ni, Fe) for water splitting: recent progress and future challenges. Energ. Environ. Sci..

[21] Cobo S (2012). A Janus cobalt-based catalytic material for electro-splitting of water. Nat. Mater..

[22] Lee S (2019). Subnanometer cobalt oxide clusters as selective low temperature oxidative dehydrogenation catalysts. Nat. Commun..

[23] Lai WZ (2012). Why is cobalt the best transition metal in transition-metal hangman corroles for O-O bond formation during water oxidation?. J. Phys. Chem. Lett..

[24] Smith PF (2014). What determines catalyst functionality in molecular water oxidation? Dependence on ligands and metal nuclearity in cobalt clusters. Inorg. Chem..

[25] Pembere AMS, Liu X, Ding W, Luo Z (2018). How partial atomic charges and bonding orbitals affect the reactivity of aluminum clusters with water?. J. Phys. Chem. A.

[26] Reber AC, Khanna SN, Roach PJ, Woodward WH, Castleman AW (2010). Reactivity of aluminum cluster anions with water: origins of reactivity and mechanisms for H2 release. J. Phys. Chem. A.

[27] Roach PJ, Woodward WH, Castleman AW, Reber AC, Khanna SN (2009). Complementary active sites cause size-selective reactivity of aluminum cluster anions with water. Science.

[28] Luo Z, Smith JC, Woodward WH, Castleman AW (2012). Reactivity of aluminum clusters with water and alcohols: competition and catalysis?. J. Phys. Chem. Lett..

[29] Zhang H (2020). Hydrogen release from a single water molecule on V_n_^+^ (3 ≤ N ≤ 30). Commun. Chem..

[30] Zhang H (2021). Vanadium cluster neutrals reacting with water: superatomic features and hydrogen evolution in a fishing mode. J. Phys. Chem. Lett..

[31] Lower, S. Activity series of metals. https://chem.libretexts.org/ (2012).

[32] Wu H (2018). Ultrafast deep-ultraviolet laser ionization mass spectrometry applicable to identify phenylenediamine isomers. Anal. Chem..

[33] Zhang H (2019). An integrated instrument of DUV-IR photoionization mass spectrometry and spectroscopy for neutral clusters. Rev. Sci. Instrum..

[34] Geng L (2021). Co_13_O_8_—metalloxocubes: a new class of perovskite-like neutral clusters with cubic aromaticity. Natl Sci. Rev..

[35] Geng L (2020). Reactivity of cobalt clusters Co_n_^+/-/0^ with ammonia: Co_3_^+^ cluster catalysis for NH_3_ dehydrogenation. J. Phys. Chem. A.

[36] Francisco H, Bertin V, Soto JR, Castro M (2016). Charge and geometrical effects on the catalytic N_2_O reduction by Rh_6_^–^ and Rh_6_^+^ clusters. J. Phys. Chem. C..

[37] Fielicke A (2004). Size and charge effects on the binding of CO to small isolated rhodium clusters. J. Phys. Chem. B.

[38] Henry DJ, Yarovsky I (2009). Dissociative adsorption of hydrogen molecule on aluminum clusters effect of charge and doping. J. Phys. Chem. A.

[39] Yang M, Zhang H, Jia Y, Yin B, Luo Z (2020). Charge-sensitive cluster−π interactions cause altered reactivity of Al_n_^±,0^ clusters with benzene: enhanced stability of Al_13_^+^Bz. J. Phys. Chem. A.

[40] Wu D-Y (2008). Theoretical study of binding interactions and vibrational raman spectra of water in hydrogen-bonded anionic complexes: (H_2_O)_N_^−^ (n = 2 and 3), H_2_OX^−^ (X = F, Cl, Br, and I), and H_2_OM (M = Cu, Ag, and Au). J. Phys. Chem. A.

[41] Alvarez-Barcia S, Flores JR (2012). Size, adsorption site, and spin effects in the reaction of Al clusters with water molecules: Al_17_ and Al_28_ as examples. J. Phys. Chem. A.

[42] Whitman WG (1926). Corrosion of iron. Chem. Rev..

[43] All-About-Chart. Corrosion resistance chart for metals. https://alexakhtar.z19.web.core.windows.net/corrosion-resistance-chart-for-metals.html (2023).

[44] Zhang H (2019). Furthering the reaction mechanism of cationic vanadium clusters towards oxygen. Phys. Chem. Chem. Phys..

[45] Grimme S, Antony J, Ehrlich S, Krieg H (2010). A consistent and accurate ab initio parametrization of density functional dispersion correction (DFT-D) for the 94 elements H-Pu. J. Chem. Phys..

[46] Frisch, M. et al. *Gaussian 16, Revision A. 03, Gaussian, Inc., Wallingford CT* (2016).

[47] Florian W, Reinhart A (2005). Balanced basis sets of split valence, triple zeta valence and quadruple zeta valence quality for H to Rn: design and assessment of accuracy. Phys. Chem. Chem. Phys..

[48] Lu T, Chen F (2012). Multiwfn: a multifunctional wavefunction analyzer. J. Comput. Chem..

[49] Humphrey W, Dalke A, Schulten K (1996). VMD: visual molecular dynamics. J. Mol. Graph..

