# Supplementary material for: On the nature of Con±/0 clusters reacting with water and oxygen
Source: Commun Chem. 2024 Mar 30;7:68. doi: 10.1038/s42004-024-01159-6 (PMC10981683; doi:10.1038/s42004-024-01159-6)
Supplement: Supplementary file 2 — Supplementary Information [file 42004_2024_1159_MOESM2_ESM.pdf]

Supporting Information for

On the nature of $Co_n^{\pm/0}$ clusters reacting with water and oxygen

Lijun Geng,[a,#] Pengju Wang,[b,#] Shiquan Lin,[a,c] Ruili Shi,[b] Jijun Zhao[b,d]* and Zhixun Luo[a,c]*

[a] Beijing National Laboratory for Molecular Sciences (BNLMS), State Key Laboratory for Structural Chemistry of Unstable and Stable Species, Institute of Chemistry, Chinese Academy of Sciences, Beijing 100190, P. R. China.

[b] Key Laboratory of Materials Modification by Laser, Ion and Electron Beams, Ministry of Education, Dalian University of Technology, Dalian 116024, P. R. China.

[c] University of Chinese Academy of Sciences, Beijing 100049, P. R. China.

[d] Guangdong Basic Research Centre of Excellence for Structure and Fundamental Interactions of Matter, Guangdong Provincial Key Laboratory of Quantum Engineering and Quantum Materials, School of Physics, South China Normal University, Guangzhou 510006, P. R. China

[#]These authors contributed equally to this work.

*Email: zhaojj@scnu.edu.cn; zxluo@iccas.ac.cn

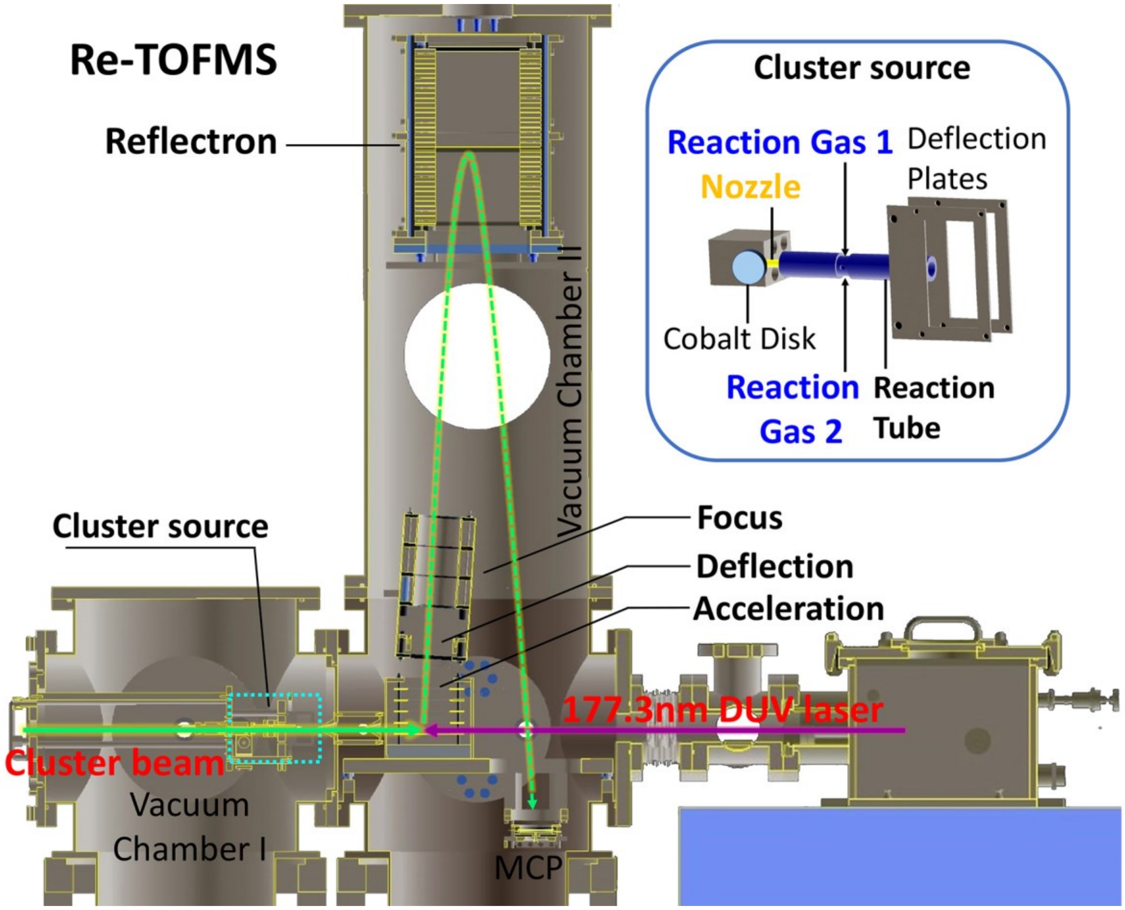

Fig. S1 Experimental instrumentation. A sketch showing the home-made reflection time-of-flight mass spectrometer (Re-TOFMS), combined with the customized deep ultraviolet 177.3 nm ps-pulsed laser for photoionization.

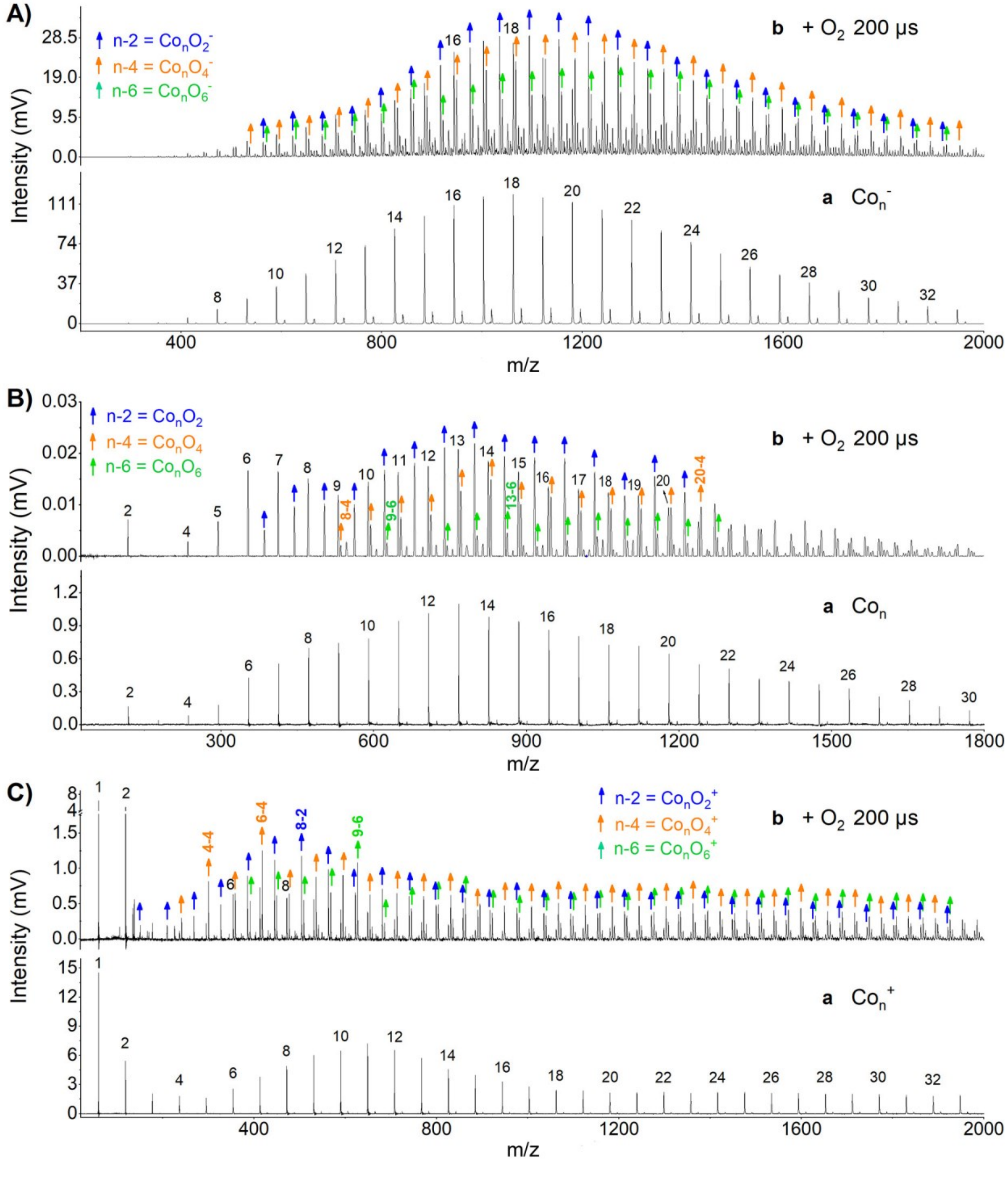

Fig. S2 Typical mass spectra of the anionic Co_n^- (A), neutral Co_n **(B)**, and cationic Co_n^+ **(C)** clusters and their reactions with O_2 (20% in He) in the same flow tube reactor. The neutral clusters are ionized by a ps-pulsed deep UV laser at 177.3nm.

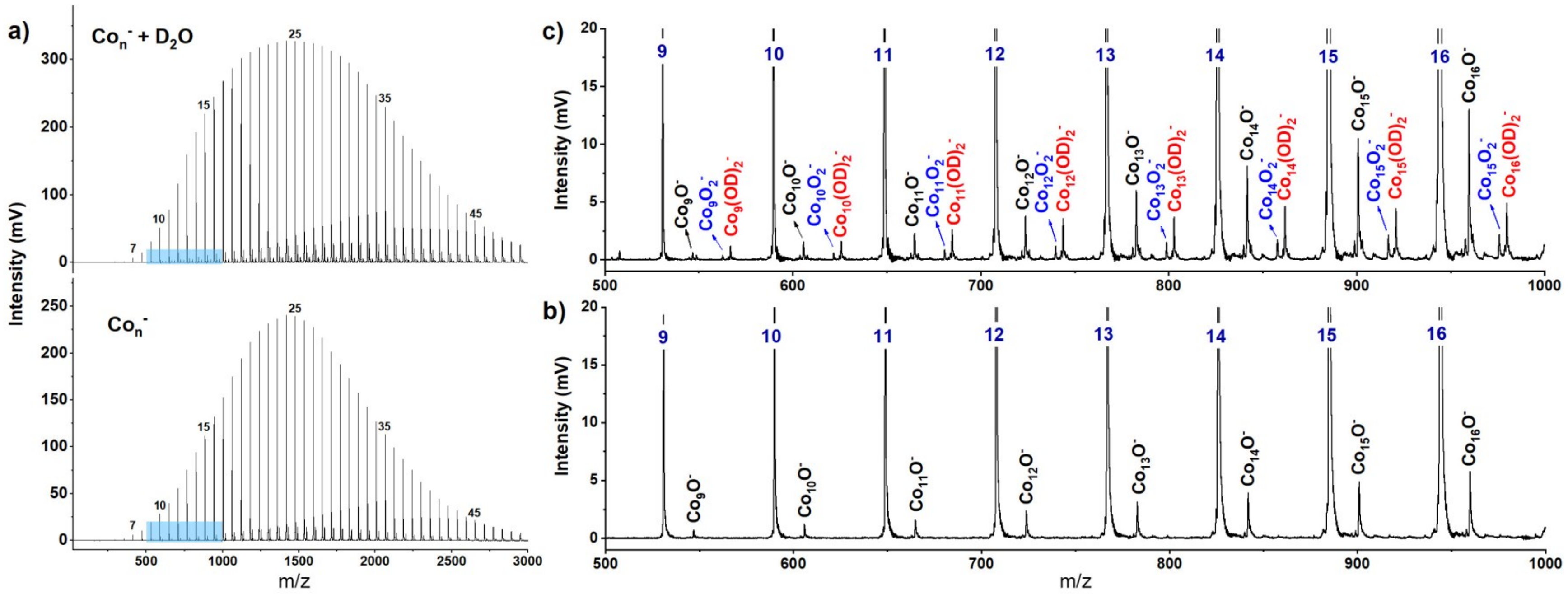

Fig. S3 Reaction with D_2O. Typical mass spectra of **(a)** the Co_n^- clusters and the reaction products with D_2O being introduced into the flow tube. The blue transparent area corresponds to the enlarged area in **(b)** and **(c)**.

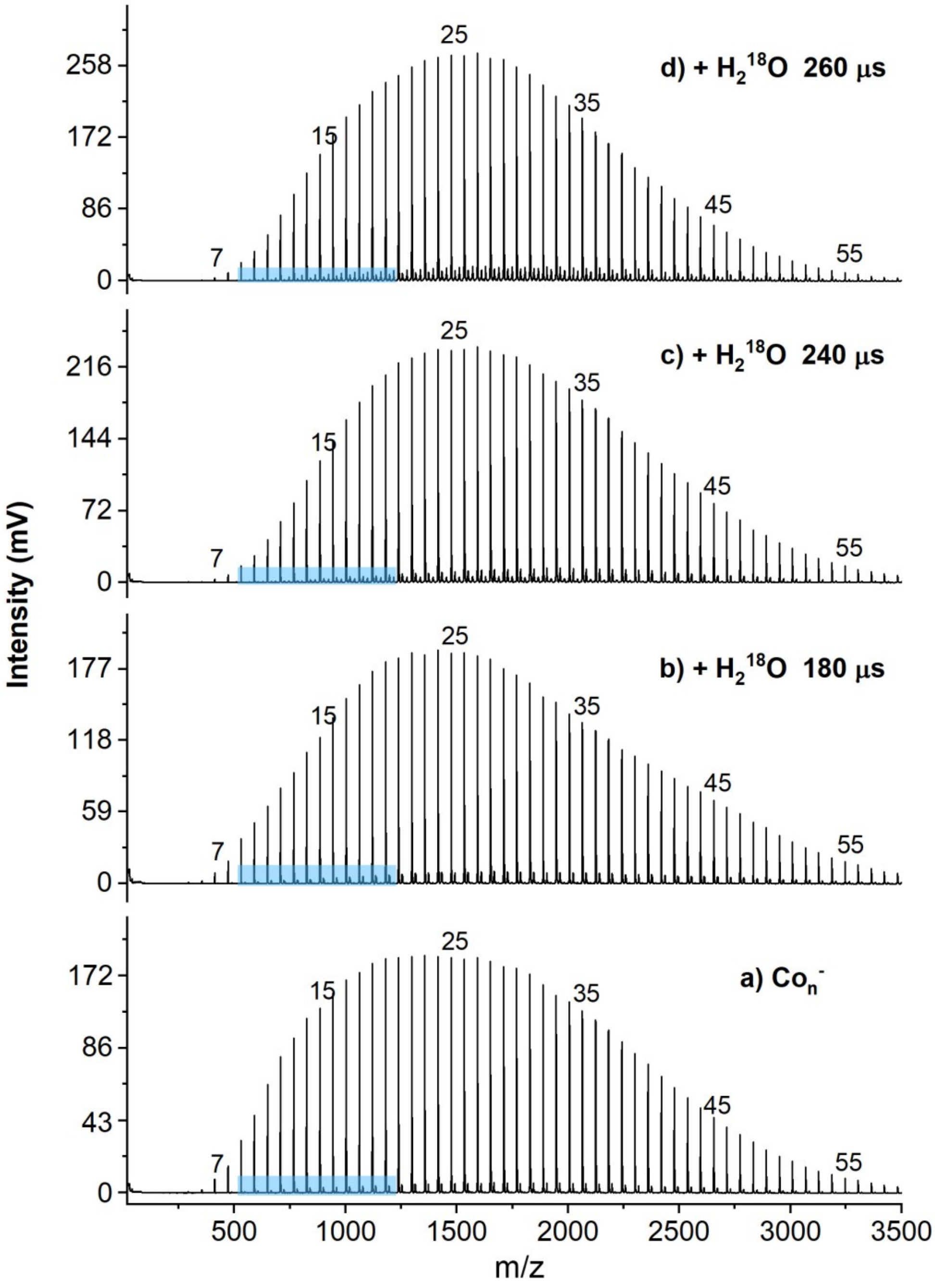

Fig. S4 Reaction with $H_2^{18}O$. Typical mass spectra of **(a)** the Co_n^- clusters produced via the homemade LaVa source within a 35 mm nozzle under 10.0 atm gas pressure, and **(b-d)** the reaction products with different amounts of $H_2^{18}O$ being introduced into the flow tube, controlled by a pulsed valve with varying pulse widths at 180 μs, 240 μs, and 260 μs, respectively. The blue transparent area corresponds to the enlarged area in Fig. 2.

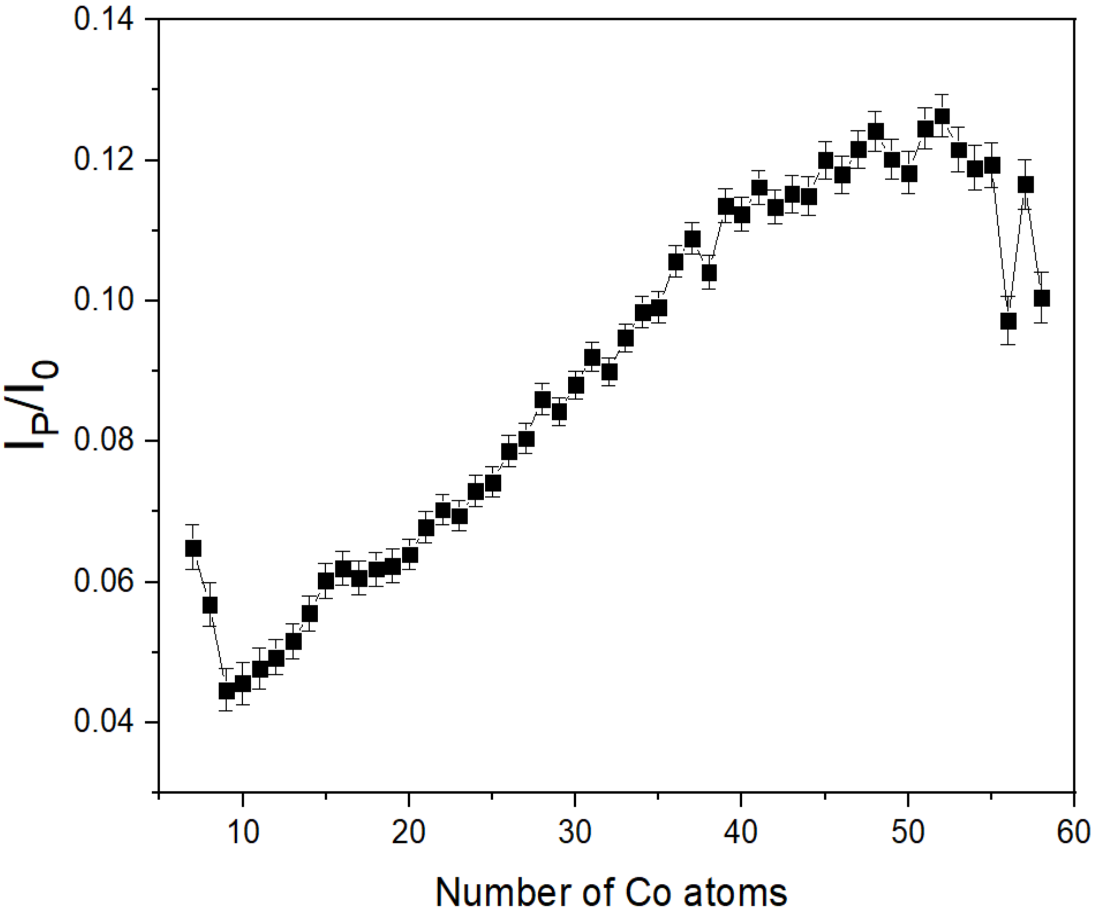

Fig. S5 Experimentally determined relation reactivity of Co_n^- (n = 7-58) with $H_2{}^{18}O$. The I_p and I_0 represent the product ions intensity and the sum of the ion intensities of all the correlative reactants and products, respectively. These value plots are based on integrated intensities of Fig. S2b (180 μs pulse width of $H_2{}^{18}O$). The error bars show the uncertainty range of values.

The ratio of the product intensity (I_p) to the total ion intensity (I_0, a sum of all the correlative reactants and products) indicates that the reactivity becomes progressively stronger as the cluster size increases except for $Co_{7,8}^-$, which becomes gradually weakened at $n > 50$, indicating altered reactivity for the relatively small-sized cobalt clusters (e.g., $n < 50$) and large ones (e.g., $n > 50$).

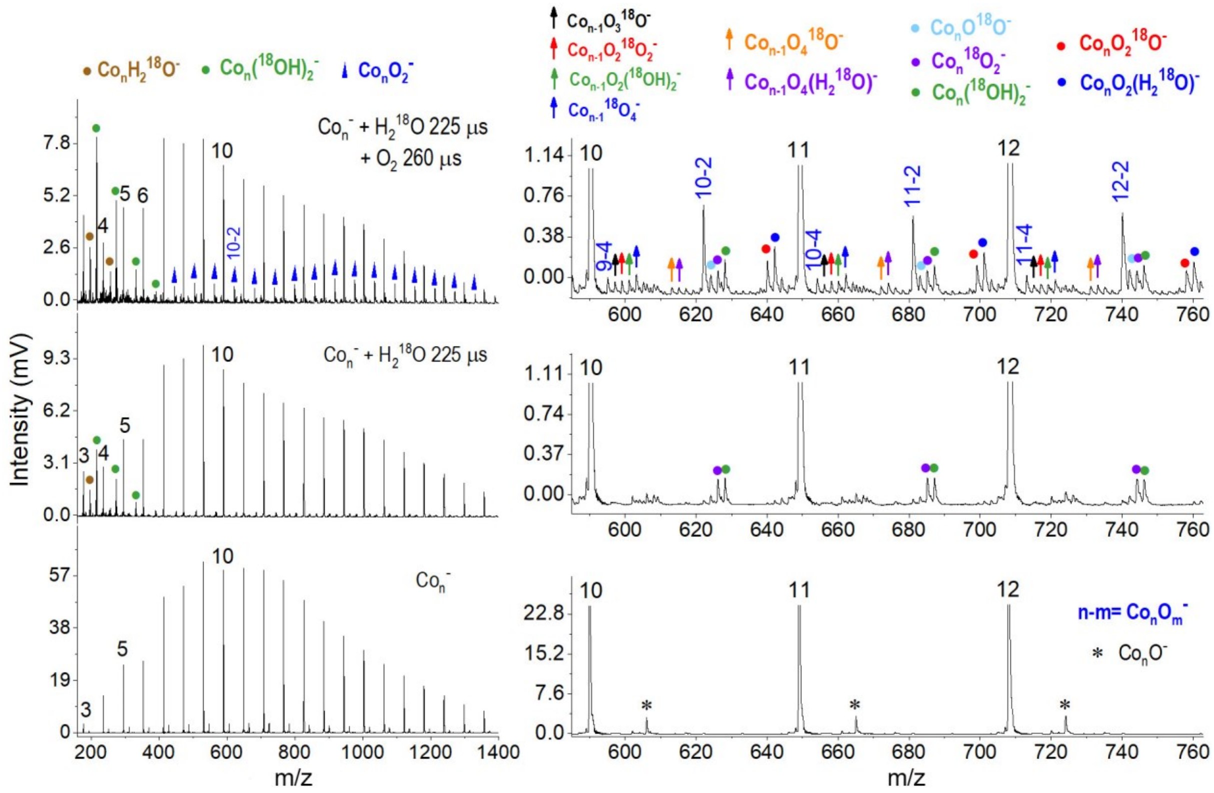

Fig. S6 Co_n^- reacting with H_2O and then O_2. Typical mass spectra of the anionic Co_n^- (n = 3-23) clusters and after their reactions with $H_2{}^{18}O$, and then both O_2 and $H_2{}^{18}O$ in the flow tube, controlled by two pulse valves.

As given in Fig. 4 of the main text, when the Co_n^- clusters react with oxygen first, diverse $Co_nO_{2x}^-$ clusters will be formed with rare exception. As a comparison, we also conducted the experiments with water to be introduced first, as shown in Fig. S6, where the weak peaks marked with purple and green correspond to minor dehydrogenation products. In the presence of both water and oxygen, Co_n^- clusters undergo both dehydrogenation and oxidation reactions, that is, the dehydrogenated $Co_n(OH)_x^-$ do not render inhibiting effect for the oxidation.

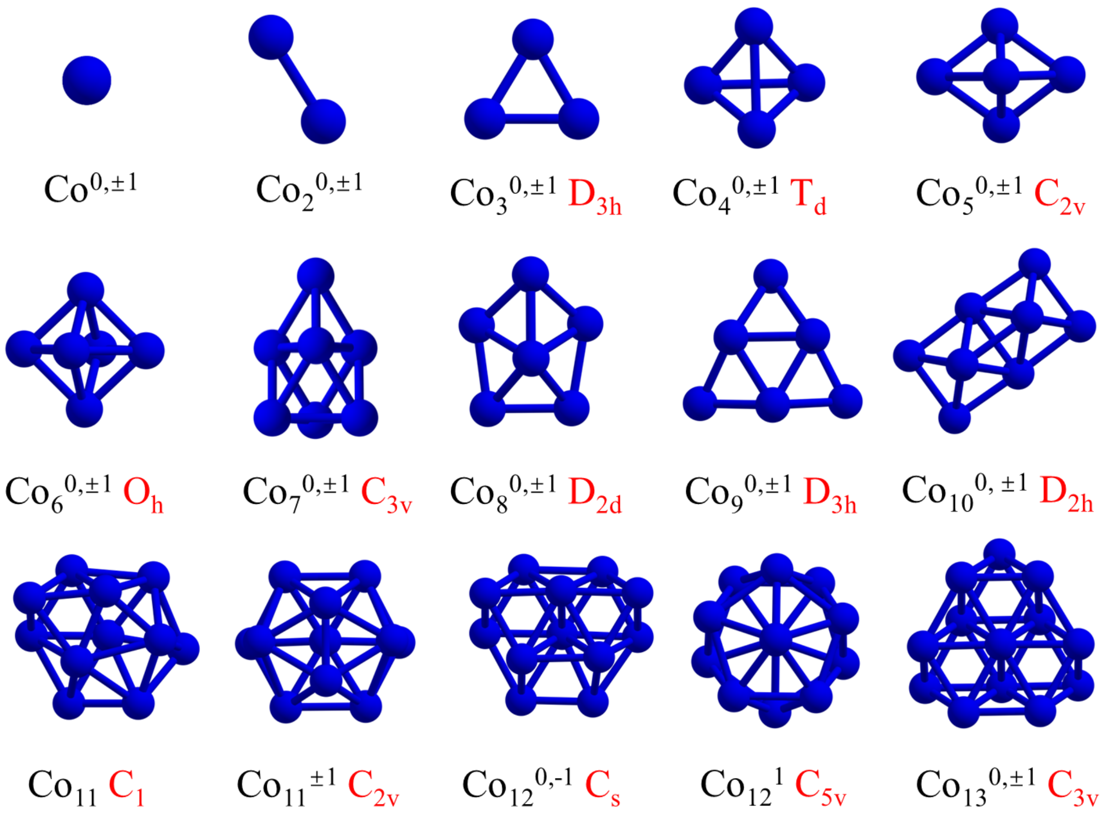

Fig. S7 The global minimum structures of Co$_n^{\pm/0}$ (n = 1-13) clusters by DFT calculations at the PBE-D3/def2-tzvp level of theory.

Table S1. The spin multiplicities of the global minimum structure of Co$_n^{\pm/0}$ clusters.

	Spin multiplicity		
n	Cationic	Neutral	Anionic
1	3	4	3
2	6	5	6
3	7	8	9
4	8	11	10
5	11	14	13
6	16	15	14
7	17	16	15
8	18	17	16
9	19	20	17
10	20	19	22
11	21	22	23
12	22	25	26
13	25	28	27

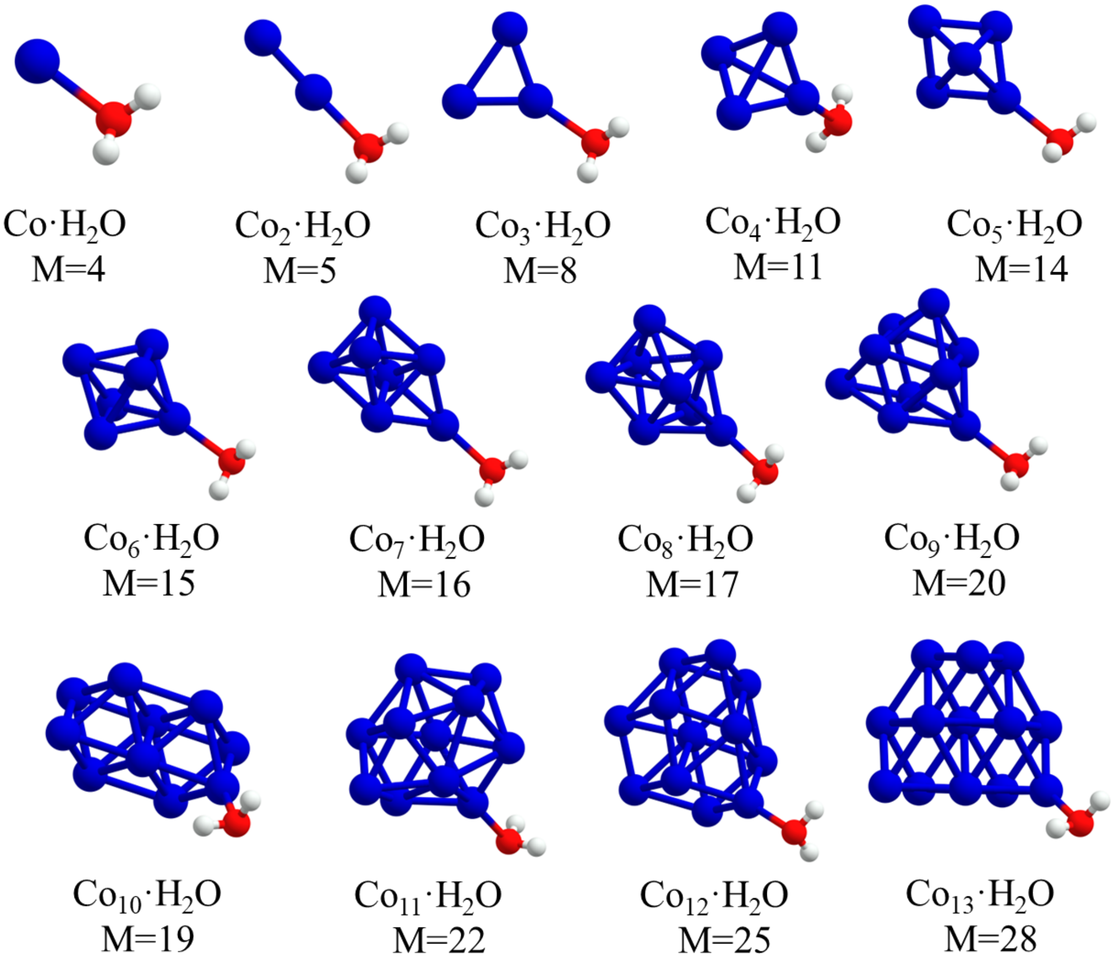

Fig. S8 The global minimum structures of Co$_n$·H$_2$O (n = 1-13) clusters by DFT calculations at the PBE-D3/def2-tzvp level of theory. Letter M refers to spin multiplicities.

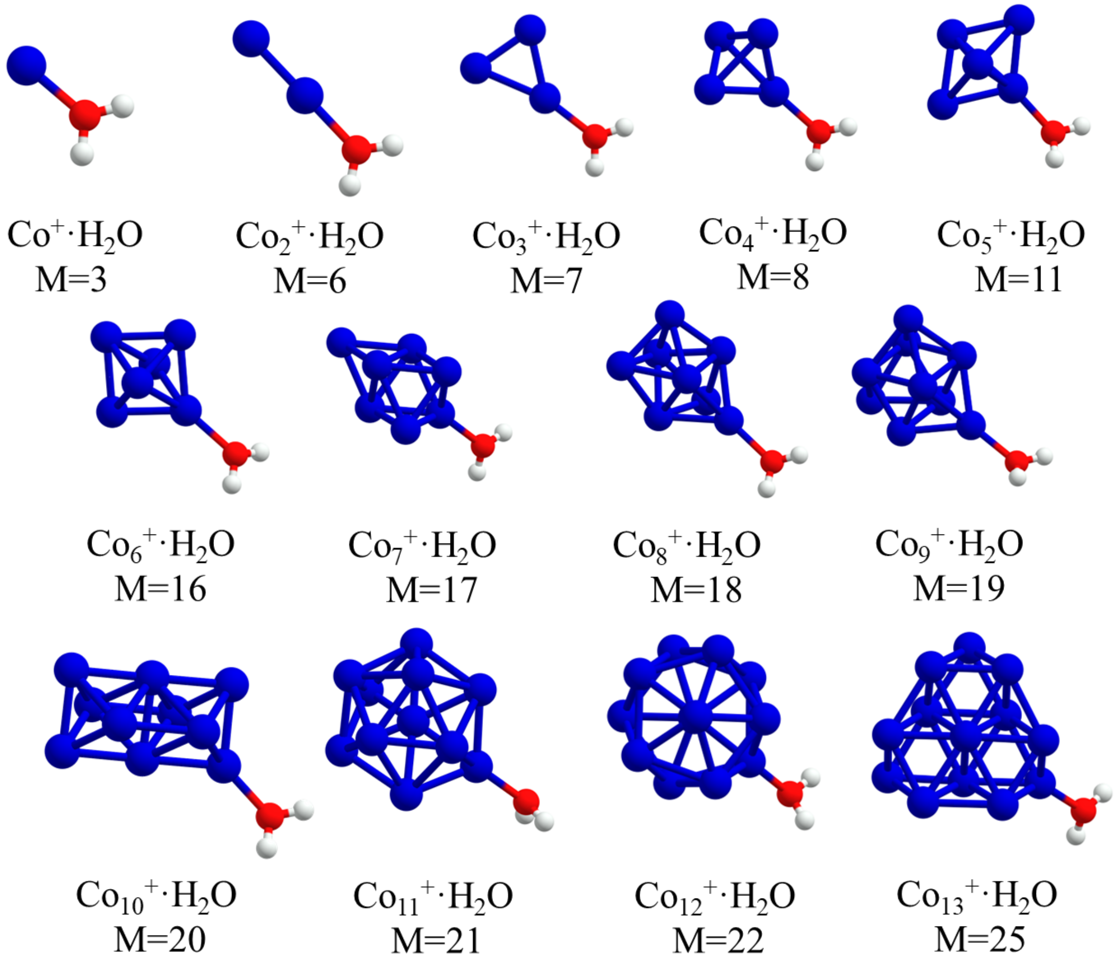

Fig. S9 Ground state structures of the $Co_n^+ \cdot H_2O$ (n = 1−13) clusters. Letter M refers to spin multiplicities.

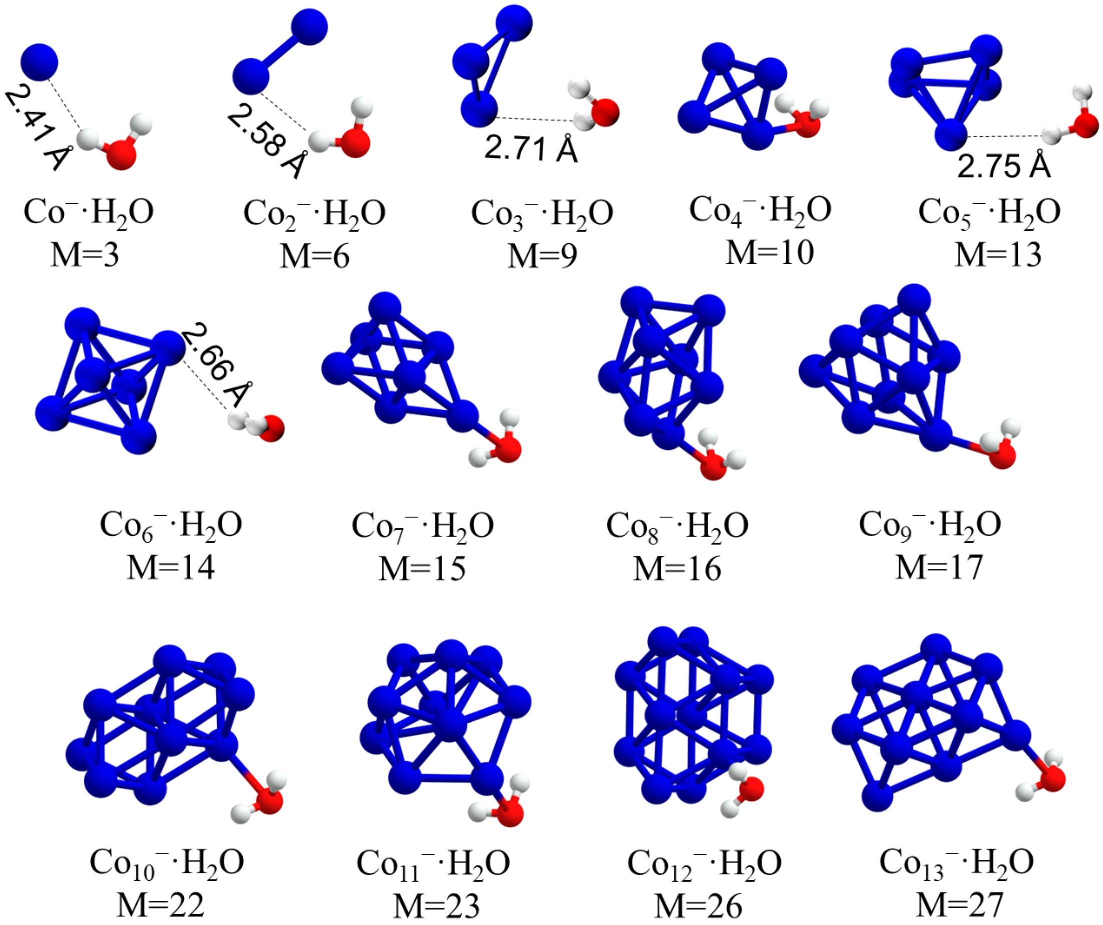

Fig. S10 Ground state structures of the $Co_n^{-}\cdot H_2O$ (n = 1−13) clusters. Letter M refers to spin multiplicities.

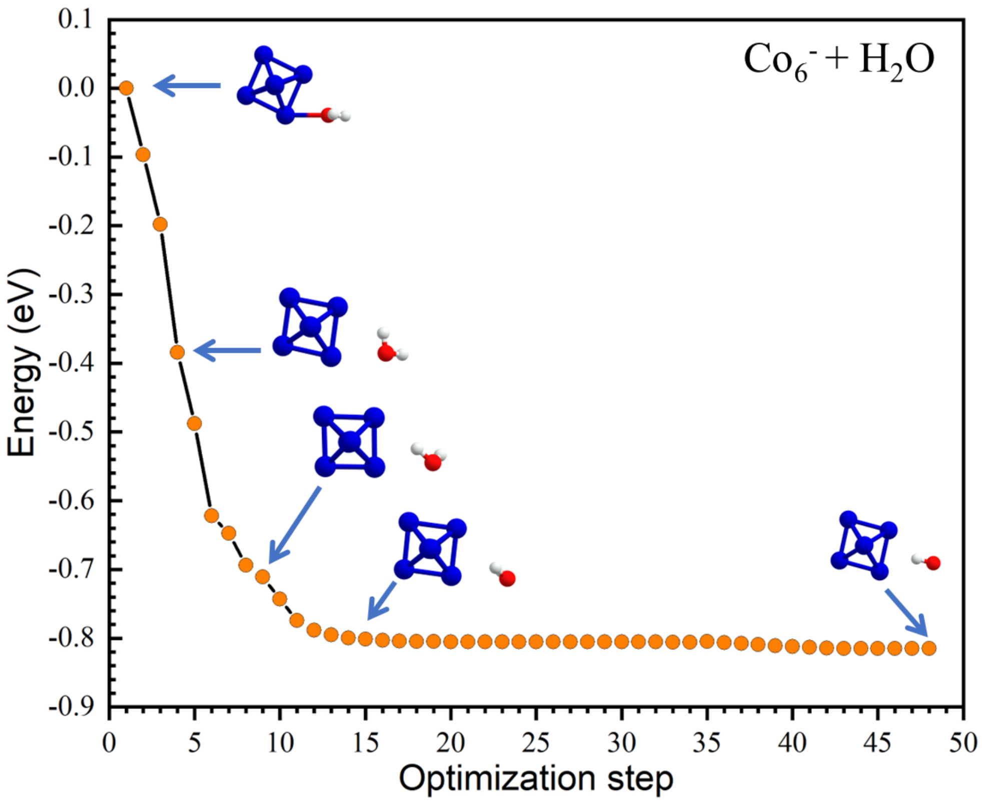

Fig. S11 The orientation of the water molecule toward the Co_6^- cluster within the optimization of the $Co_6^- \cdot H_2O$ structure.

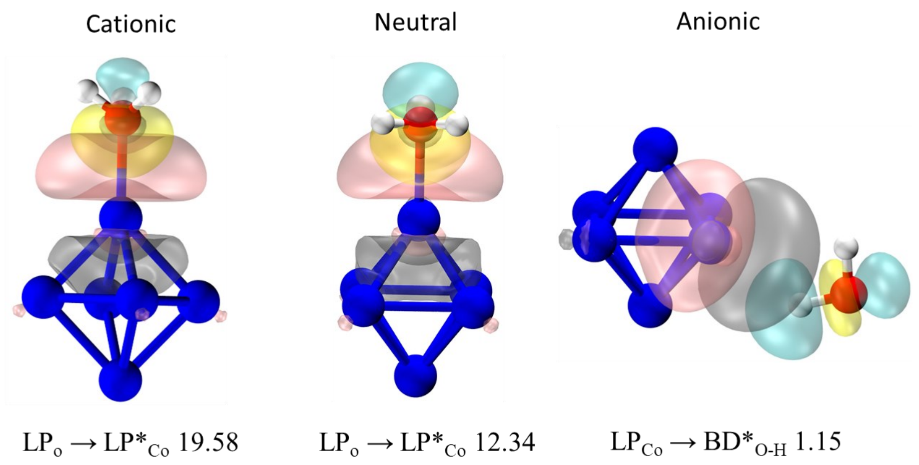

Fig. S12 Dominant natural bond orbital (NBO) donor–acceptor (overlap) interactions in $Co_6(H_2O)^{\pm/0}$ clusters. The second-order perturbative energies are given in $kcal \cdot mol^{-1}$.

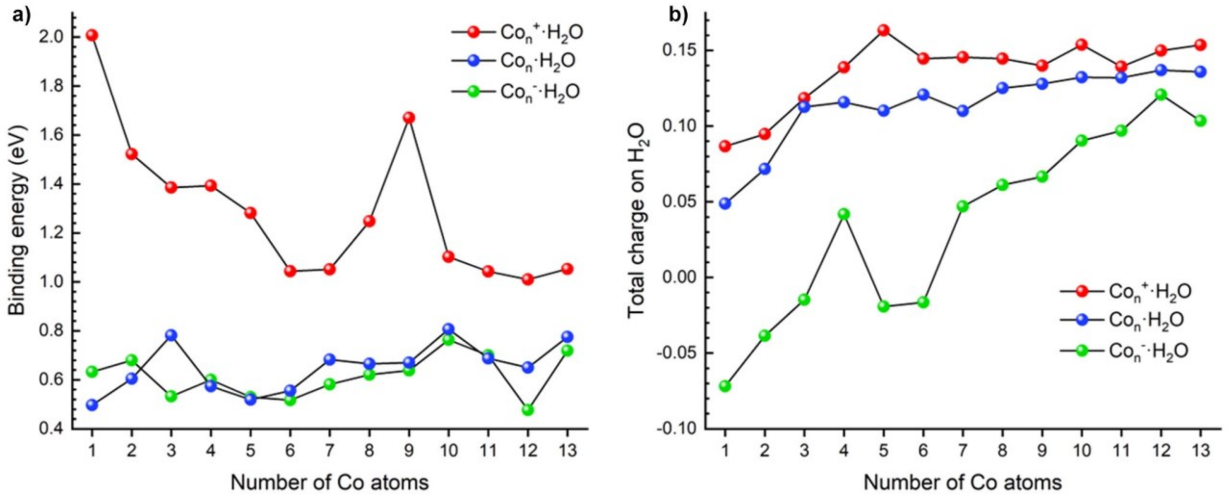

Fig. S13 Energetics. (a) Biding energy of $Co_n^{\pm/0}$ with one H_2O molecule (a), defined as E_{ad} = $E(Co_n^{\pm/0}\cdot H_2O)$ - $E(Co_n^{\pm/0})$ - $E(H_2O)$. **(b)** NPA charges on H_2O for the $Co_n^{\pm/0}\cdot H_2O$. Energies are given in eV.

To unveil the charge effect of cobalt clusters interacting with H_2O, we examined the total natural population analysis (NPA) charge distributions (Fig. S13) on the H_2O molecules for all the $Co_n^{\pm/0}H_2O$ ($n \leq 13$) clusters. As a result, when an H_2O molecule is adsorbed on the $Co_n^{\pm/0}$ clusters, the negative NPA charges mainly distribute on the cobalt showing minor electron transfer.

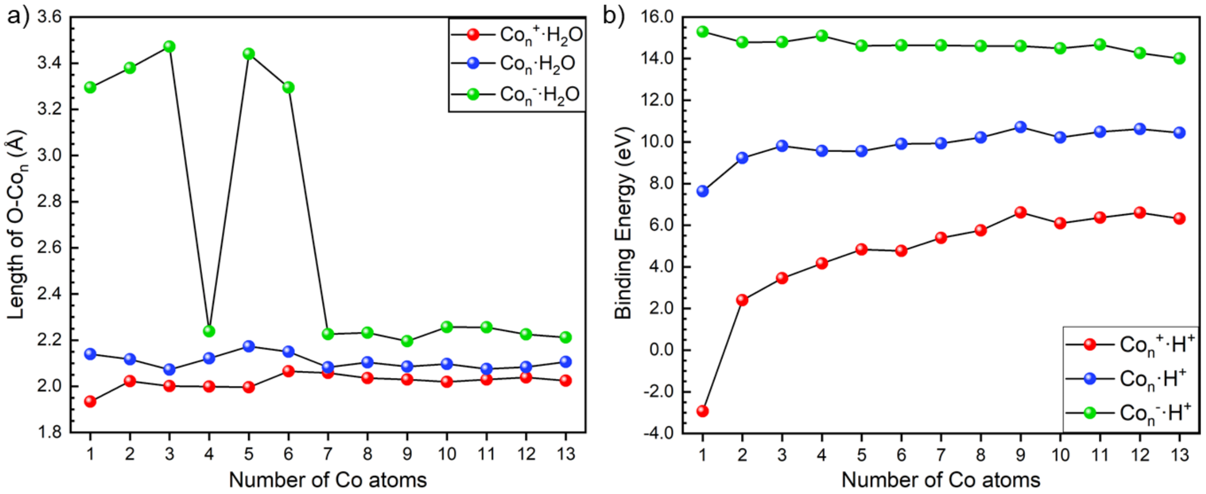

Fig. S14 Bond length and binding energy. (a) The O-Co bond lengths/distance in the $Co_n^{\pm/0}\cdot H_2O$ clusters. **(b)** Proton affinity energies of the $Co_n^{\pm/0}$ clusters. Energies are given in eV.

Table S2 Gibbs Free Energies. Biding energy of $Co_n^{\pm/0}$ with one H_2O molecule and proton affinity energies of the $Co_n^{\pm/0}$ clusters. Energies are given in eV.

	Biding energy of $Co_n^{\pm/0}$ with H_2O			Proton affinity energies (eV)		
n	cation	neutral	anion	cation	neutral	anion
1	1.44	0.27	0.44	-3.10	7.44	15.11
2	1.24	0.32	0.45	2.18	9.04	14.59
3	1.06	0.45	0.26	3.22	9.54	14.51
4	1.01	0.25	0.24	3.90	9.32	14.83
5	0.88	0.21	0.23	4.58	9.29	14.34
6	0.71	0.18	0.22	4.51	9.64	14.37
7	0.73	0.40	0.22	5.14	9.75	14.35
8	0.85	0.31	0.24	5.46	9.91	14.33
9	1.34	0.37	0.31	6.34	10.45	14.31
10	0.85	0.75	0.41	5.85	10.25	14.23
11	0.69	0.45	0.30	6.12	10.32	14.34
12	0.63	0.51	0.35	6.34	10.54	14.27
13	0.70	0.31	0.22	6.03	10.17	13.76

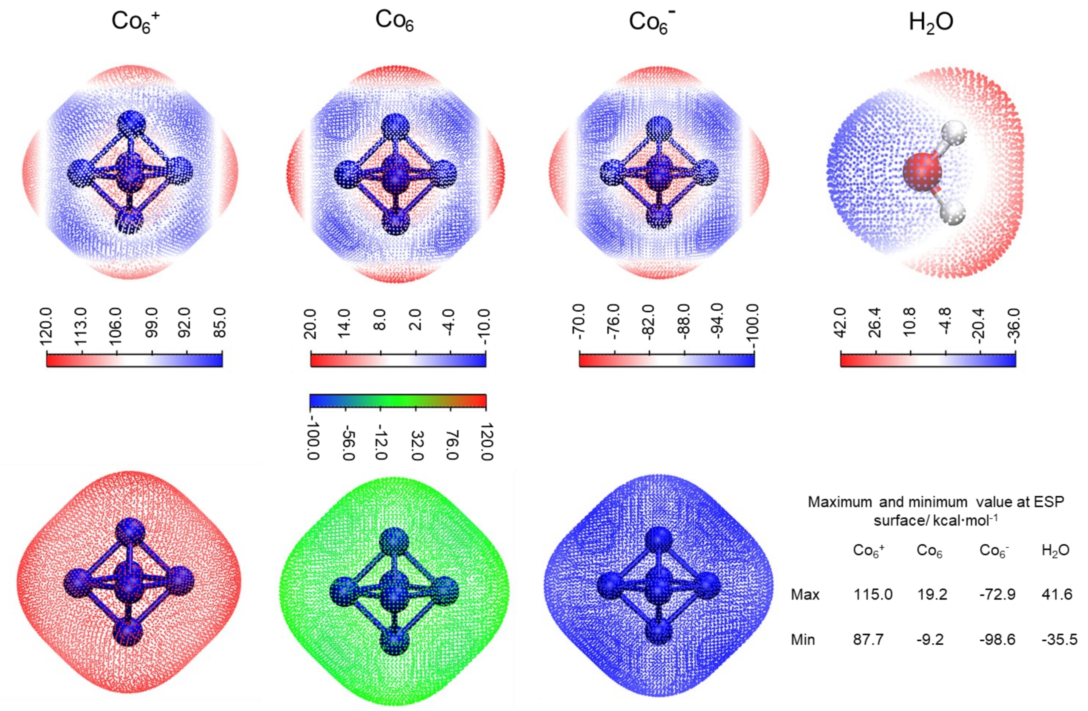

Fig. S15 The global value on the electrostatic potential (ESP) mapped the molecular surface of $Co_6^{\pm/0}$ clusters and H_2O molecule. The unit is in kcal·mol^{-1}. The electron-rich density regions are mapped with blue colour, and the electron-poor regions are mapped with red colour.

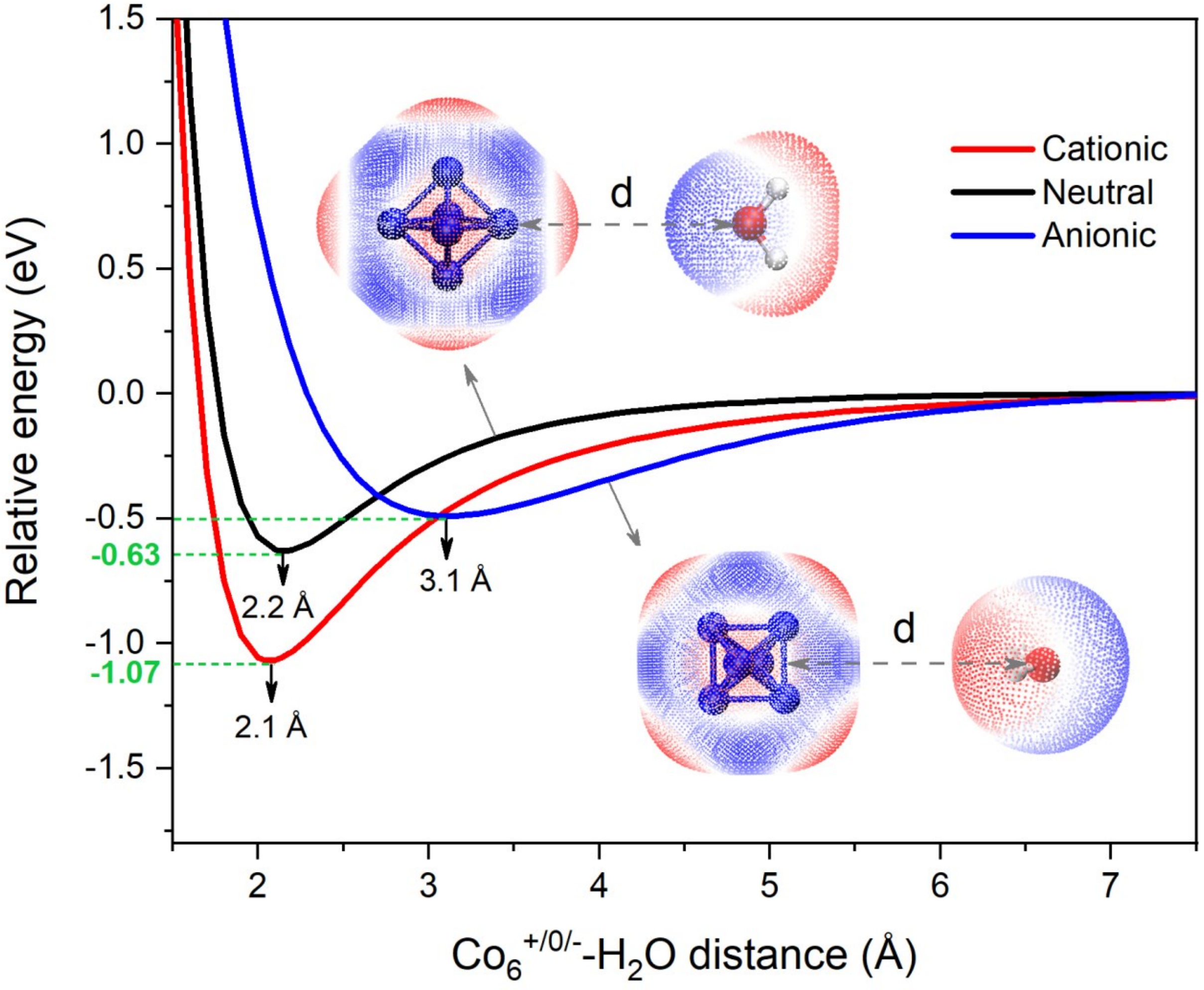

Fig. S16 Potential scan showing the relative energy of $H_2O{\cdot}Co_6^{\pm/0}$ relative to the approaching distance between H_2O and the $Co_6^{\pm/0}$ clusters.

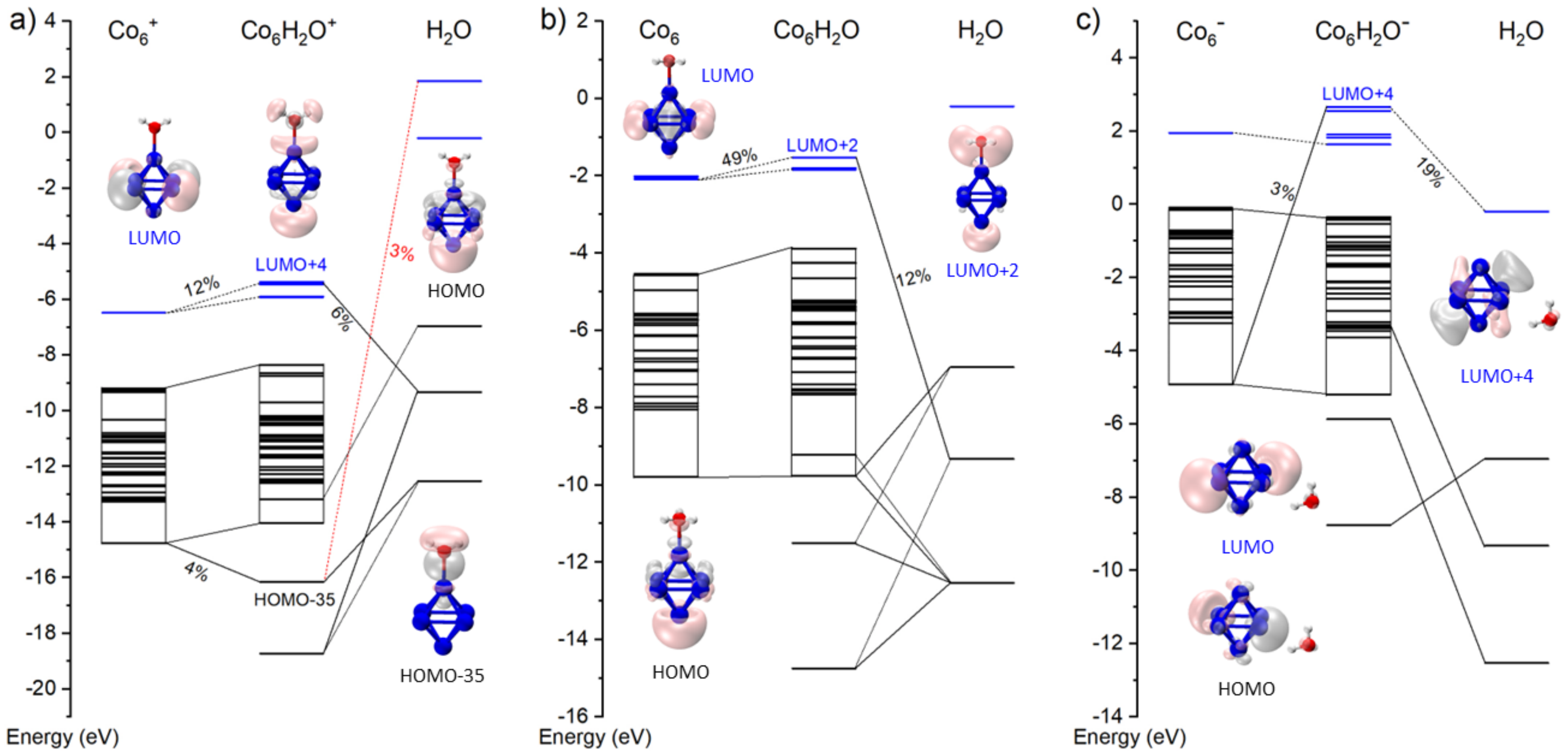

Fig. S17 Molecular orbital interaction diagram of $Co_6(H_2O)^{\pm/0}$ based on the charge decomposition analysis (CDA). The blue and black lines represent the unoccupied and occupied orbitals, respectively. Dashed lines indicate the correlations between the fragment orbitals ($Co_6^{\pm/0}$, H_2O) and the $Co_6(H_2O)^{\pm/0}$ cluster orbitals, with the insets showing the frontier molecular orbital. Fragment 1: Co_5^{+}; Fragment 2: adsorbed H_2O molecule.

Table S3 Thermodynamics energy changes of $Co_{1,6}^{\pm/0}$ clusters reacting with one or two H_2O molecules accompanied by hydrogen generation. Energies are given in eV.

	ΔE (eV)	ΔG (eV)
$Co_1^+ + H_2O \rightarrow Co_1O^+ + H_2$	2.03	2.04
$Co_1 + H_2O \rightarrow Co_1O + H_2$	0.31	0.31
$Co_1^- + H_2O \rightarrow Co_1O^- + H_2$	0.09	0.09
$Co_6^+ + H_2O \rightarrow Co_6O^+ + H_2$	-0.06	0.08
$Co_6 + H_2O \rightarrow Co_6O + H_2$	-0.12	0.02
$Co_6^- + H_2O \rightarrow Co_6O^- + H_2$	-0.32	-0.05
$Co_6^+ + 2H_2O \rightarrow Co_6(OH)_2^+ + H_2$	-0.77	-0.31
$Co_6 + 2H_2O \rightarrow Co_6(OH)_2 + H_2$	-1.54	-1.07
$Co_6^- + 2H_2O \rightarrow Co_6(OH)_2^- + H_2$	-2.05	-1.59

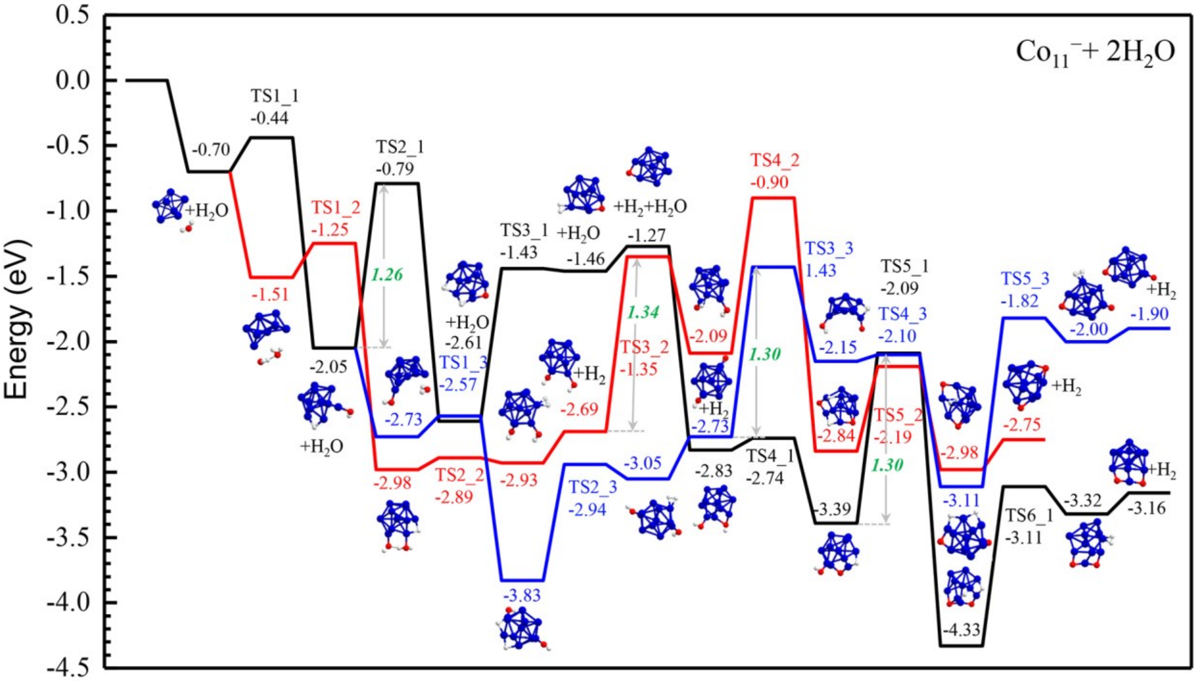

Fig. S18 Reaction coordinates of Co_{11}^-. The reaction energy diagram of "$Co_{11}^- + 2\ H_2O \rightarrow Co_{11}O^- + H_2 + H_2O \rightarrow Co_{11}O_2^- + 2\ H_2$", "$Co_{11}^- + 2\ H_2O \rightarrow Co_{11}(OH)_2^- + H_2$" and "$Co_{11}^- + 2\ H_2O \rightarrow Co_{11}O_2^- + 2\ H_2$". All the energies are given in eV.

We calculated the reaction coordinates of "Co_{11}^- + H_2O" and "Co_{11}^- + 2 H_2O" as depicted in Fig. S18. The reaction path of "Co_{11}^- + H_2O → $Co_{11}O^-$ + H_2" shows a rate-determining step energy barrier of 1.26 eV (TS2_1) but is exothermicity (ΔE=1.27 eV), indicating both kinetic- and thermodynamic-favourable reaction pathway. Interestingly, the reaction of "Co_{11}^- + 2 H_2O → $Co_{11}(OH)_2^-$ + H_2" shows a much smaller energy barrier (0.26 eV) for the H-atom transfer (TS1_2) than the rate-determining step for a complete dehydrogenation (1.34 eV for TS3_2), which is perfectly compatible with the experimental observation that $Co_n(OH)_2^-$ show relatively larger mass abundances than that of $Co_nO_2^-$.

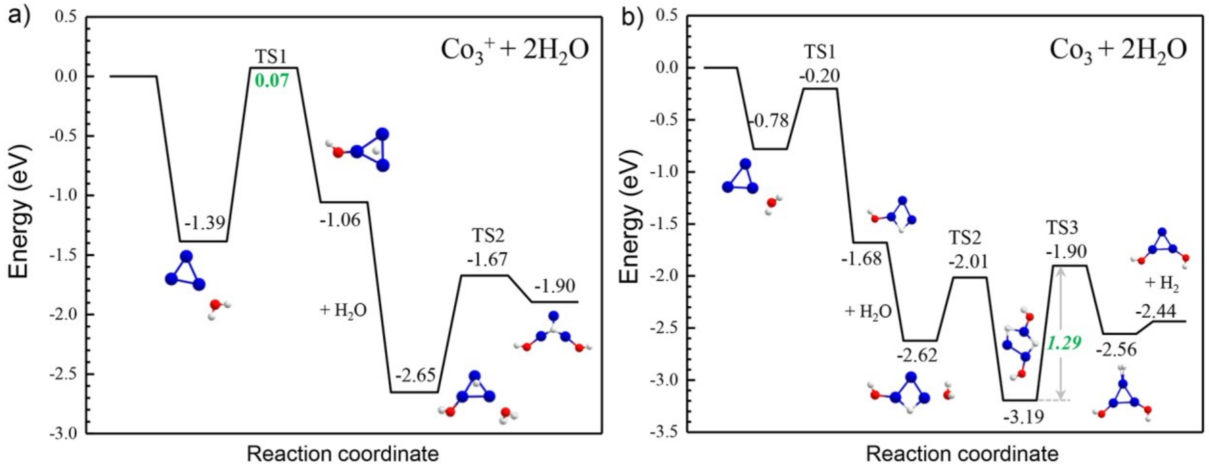

Fig. S19 Reaction coordinates of $Co_3^{+/0}$. The reaction energy diagram of the $Co_3^{+/0}$ clusters with two H_2O molecules. Energies are given in eV.

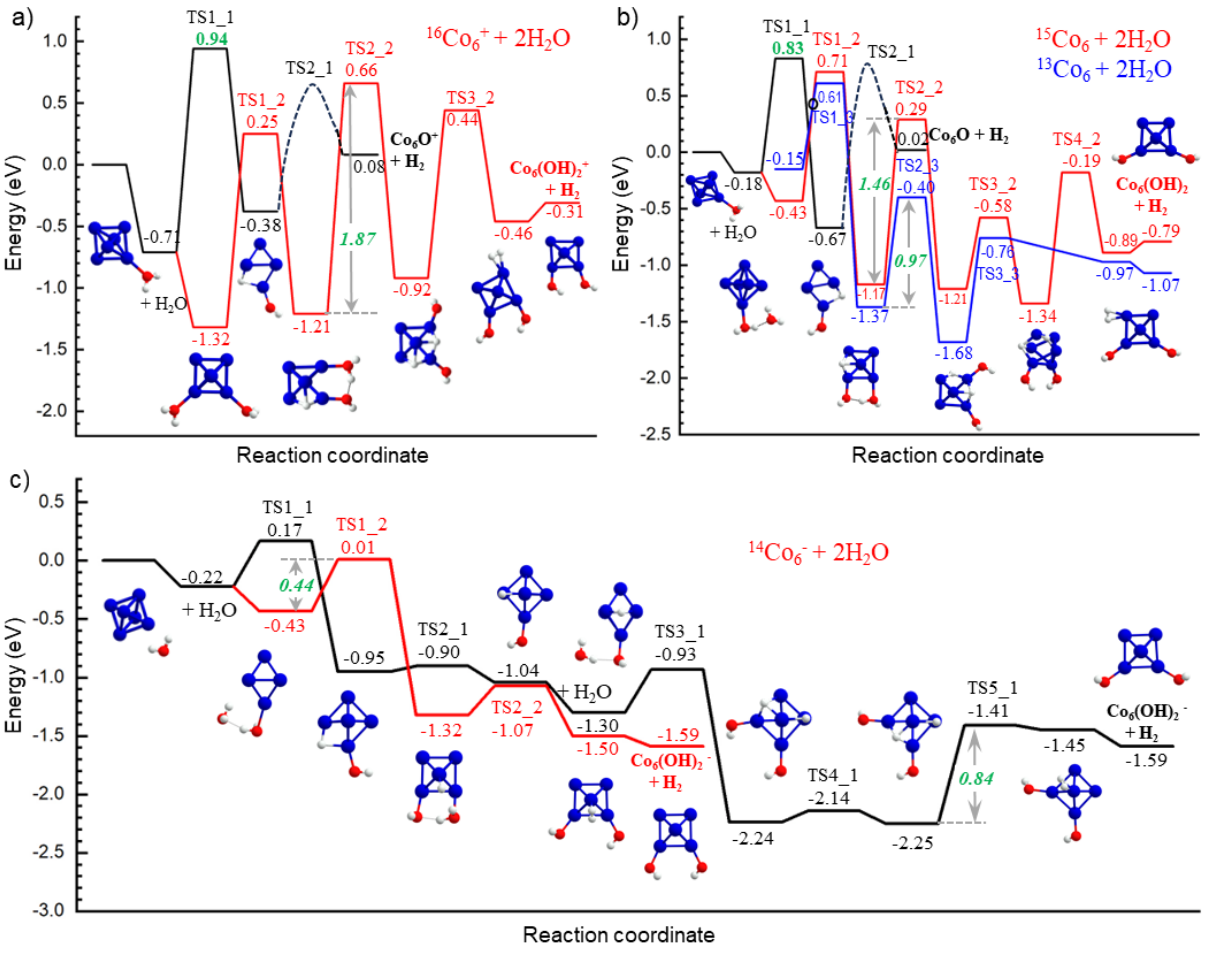

Fig. S20 Gibbs free energies of the reaction coordinates of $Co_6^{\pm/0}$ with $2H_2O$. a-c The energy diagram for cationic $^{16}Co_6^+$, anionic $^{14}Co_6^-$, and neutral $^{15}Co_6$ clusters in reacting with one and two water molecules. Energies are given in eV.
